# RepliChrom: Interpretable machine learning predicts cancer‐associated enhancer‐promoter interactions using DNA replication timing

**DOI:** 10.1002/imt2.70052

**Published:** 2025-05-27

**Authors:** Fuying Dao, Benjamin Lebeau, Crystal Chia Yin Ling, Mi Yang, Xueqin Xie, Melissa Jane Fullwood, Hao Lin, Hao Lyu

**Affiliations:** ^1^ Department of Clinical Laboratory, Sichuan Clinical Research Center for Cancer, Sichuan Cancer Hospital & Institute, Sichuan Cancer Center, School of Life Science and Technology University of Electronic Science and Technology of China Chengdu China; ^2^ School of Biological Sciences Nanyang Technological University Singapore Singapore; ^3^ The Clinical Hospital of Chengdu Brain Science Institute, School of Life Science and Technology University of Electronic Science and Technology of China Chengdu China; ^4^ Institute of Molecular and Cell Biology Agency for Science, Technology and Research (A*STAR) Singapore Singapore

## Abstract

RepliChrom is an interpretable machine learning model that predicts enhancer‐promoter interactions using DNA replication timing across multiple cell types. By integrating replication timing with chromatin interaction data from multiple experimental platforms, it accurately distinguishes true interactions and reveals promoter‐region signals as key regulatory drivers. Importantly, the RepliChrom uncovers cancer‐specific chromatin patterns in leukemia, offering mechanistic insights into how replication timing shapes long‐range gene regulation in both normal and diseased genomes.

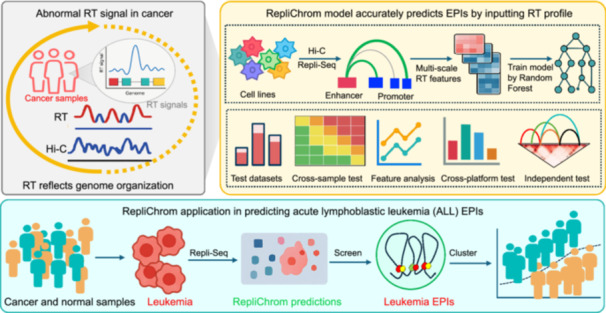

## METHODS

This study integrates Hi‐C, Hi‐TrAC, ChIA‐PET, 5C, and RT data to train the EPIs predictor RepliChrom. Full technical details are provided in the Supporting Information.

## AUTHOR CONTRIBUTIONS

**Fuying Dao**: Conceptualization; methodology; investigation; writing—original draft; writing—review and editing; software; validation; data curation. **Benjamin Lebeau**: Methodology; validation; writing—original draft; writing—review and editing. **Crystal Chia Yin Ling**: Software; validation. **Mi Yang**: Writing—original draft; writing—review and editing. **Xueqin Xie**: Writing—review and editing; writing—original draft; software. **Melissa Jane Fullwood**: Conceptualization; writing—review and editing; funding acquisition. **Hao Lin**: Writing—review and editing; funding acquisition; conceptualization. **Hao Lyu**: Conceptualization; methodology; writing—original draft; writing—review and editing; funding acquisition.

## CONFLICT OF INTEREST STATEMENT

The authors declare no conflicts of interest.

## ETHICS STATEMENT

No animals or humans were involved in this study.

To the Editor,

Chromatin interactions play a crucial role in the spatiotemporal regulation of gene [1]. They bridge distal enhancers to target genes in 3D chromatin space and form insulation domains that restrict enhancer activity [2]. High‐throughput experimental techniques, such as Chromatin Interaction Analysis with Paired‐End Tag (ChIA‐PET) [3] and High‐throughput Chromosome Conformation Capture (Hi‐C) [4], alongside their variants (Capture‐Hi‐C [5], DNase‐Hi‐C [6]), have enabled the mapping of these interactions at varying resolutions. However, these methods are costly and technically demanding. As a result, their use in large cohorts or clinical samples remains limited. Consequently, our understanding of chromatin interactions across diverse cell lines and cancers is still incomplete.

To address these challenges, computational methods have been developed. They integrate DNA sequence with various one‐dimensional regulatory signals, such as transcription factor binding and chromatin modifications to predict chromatin interactions. These approaches have greatly advanced our understanding of 3D genome organization [7]. For example, Lollipop [8] effectively predicts enhancer‐promoter interactions (EPIs) using both genomic and epigenomic features across multiple cell types. ChINN [9] applies convolutional neural networks to predict interactions between open chromatin regions from DNA sequence alone, showing strong generalization across samples. Other deep learning models, such as Akita [10] and Orca [11], predict chromatin contact maps at different genomic scales using only sequence input. MATCHA [12] further explores multiway interactions through hypergraph representation learning.

**Figure 1:**
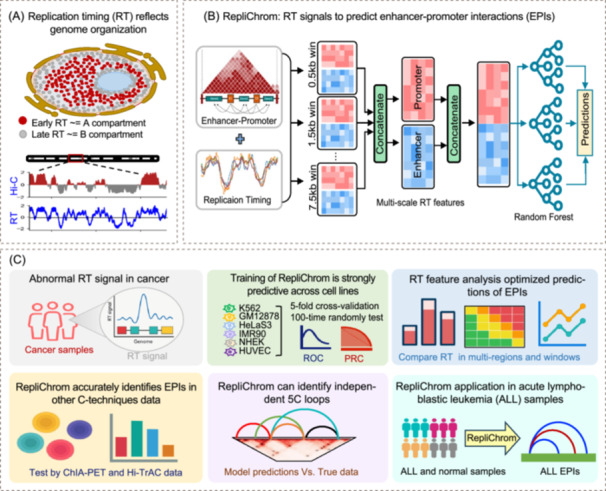
Overview of the RepliChrom model. (A) DNA replication timing (RT) reflects 3D genome organization. (B) The model takes Hi‐C loop and RT data as input, generating a data set of enhancer‐promoter interactions (EPIs) and distance‐matched non‐EPIs. Next, multi‐scale RT information was then extracted based on multi‐bins, followed by classification model training using Random Forest. (C) Analysis in this study includes RT characteristic analysis, RepliChrom model training and evaluation, RT features analysis, RepliChrom application in ChIA‐PET and Hi‐TrAC data, RepliChrom application in Chromosome Conformation Capture Carbon Copy (5C) data, and RepliChrom application in cancer.

Despite these advances, these methods all require DNA and/or other epigenetic datasets such as Chromatin Immunoprecipitation Sequencing (ChIP‐Seq) and Assay for Transposase Accessible Chromatin with high‐throughput sequencing (ATAC‐Seq), which are also laborious to produce. A simpler DNA sequence‐independent computational model would reduce computational time and costs, offer simpler implementation, and democratize its use for healthcare and large‐scale scientific investigations. Previous studies have shown that the replication timing (RT) program reflects 3D genome architecture (Figures 1A, S1) [13]. RT refers to the genome‐wide temporal order in which DNA is replicated during the S phase of the cell cycle. Early and late replicating regions are associated with active and repressive chromatin compartments, respectively. Specifically, early RT domains often coincide with gene‐rich, transcriptionally active A compartments, while late RT regions typically overlap with inactive B compartments. Building on this spatial‐temporal coupling, we hypothesize that RT may serve as an additional predictive signal for chromatin interactions, beyond conventional DNA sequence and epigenetic features.

To test this, we developed RepliChrom, a predictive framework that leverages RT signals to identify EPIs. Through rigorous in‐sample and cross‐sample evaluations, we demonstrated that RT features are predictive of EPIs and that RepliChrom is compatible across datasets from different sequencing platforms. Notably, RepliChrom enabled the discovery of cancer‐specific EPIs patterns, underscoring its potential in disease‐specific chromatin interaction analysis. In summary, RepliChrom offers a novel and interpretable approach for decoding 3D genome folding from RT, revealing both general and cancer‐specific regulatory landscapes and advancing our understanding of the DNA replication program in gene regulation.

## RESULTS AND DISCUSSION

### Abnormal RT signal across cell lines and cancer

To better characterize the potential of RT as a predictive feature, we systematically analyzed its patterns across different cell lines and among individuals within the same cell line. RT exhibits strong cell line specificity, reflecting cell‐type‐specific chromatin architecture (Figure S2A,B). Notably, aberrant RT signals in cancer samples clearly distinguish them from normal controls (Figure S2C,D), indicating that RT may serve as a potential biomarker for cancer. However, such abnormal RT patterns are less pronounced among cancer subtypes, implying more subtle differences at this level (Figure S2E,F). Furthermore, we observed a strong correspondence between RT profiles and Hi‐C interaction signals (Figure S2G–H), reinforcing the notion that RT captures key aspects of 3D genome organization. Based on these observations, we propose that computational models leveraging RT data can effectively predict EPIs.

### RepliChrom captures multi‐scale RT features

To test our hypothesis, we developed a machine learning‐based framework, RepliChrom, which uses RT profiles as the sole input to predict EPIs (Figure 1B). The model is designed to capture the spatial and temporal patterns embedded in RT data, enabling accurate EPIs prediction without relying on DNA sequence or epigenetic features.

We firstly collected raw Hi‐C data (GSE63525) of six cell lines (K562, GM12878, HeLaS3, IMR90, NHEK, and HUVEC) from GEO database [14]. To simplify analysis, adjacent enhancers or promoters within 500 bp were merged. Positive EPIs were defined by pairing these merged regions (Figure S3A), while negative EPIs were selected from non‐Hi‐C overlapping pairs, matched for distance at a 1:20 ratio (Figure S3C–H, Table S1).

For each enhancer‐promoter pair, RT features were extracted from upstream and downstream flanking regions using multiple window sizes, resulting in a 482‐dimensional multi‐scale RT feature set (Figure S3B). A Random Forest (RF) classifier was then trained and evaluated using both five‐fold cross‐validation and a 100‐time random resampling strategy, ensuring robust and reliable performance. Then, RepliChrom demonstrated robust predictive performance across multiple evaluation settings and was further applied to identify cancer‐specific EPI signatures (Figure 1C), highlighting the potential of RT for EPIs mapping even in the absence of sequence or epigenetic data.

### RepliChrom robustly predicts EPIs using only RT profiles

**Figure 2:**
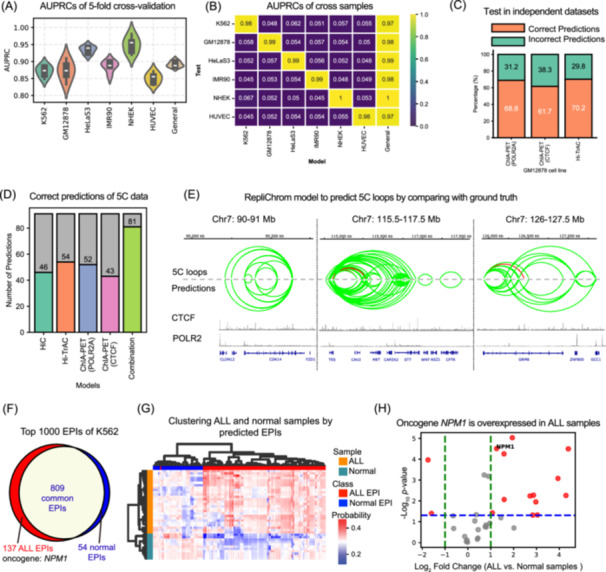
RepliChrom performance and application. (A) The AUPRCs produced by RepliChrom based on fivefold cross validation across the six cell lines and General model. (B) The heatmap of cross‐sample test on RepliChrom models. The horizontal axis is the model trained on a cell line, and the vertical axis is the test set of other cell lines. (C) Proportions of correctly predicted independent loops by the general model across different platforms, showing consistent predictive power for POLR2A/CTCF ChIA‐PET, and Hi‐TrAC datasets. (D) Summary of the number of correctly predicted 5C interactions by each individual model and the combination model. Among 91 total 5C interactions, the combination model achieves the highest recovery, demonstrating the complementary predictive power of replication timing (RT) across chromatin interaction platforms. (E) Integrated predictions from all models (combination model) show improved coverage of 5C interactions. Light green arcs represent correctly predicted loops, while red arcs denote undetected true 5C interactions. (F) Venn diagram showing the acute lymphoblastic leukemia (ALL) specific and normal specific enhancer‐promoter interactions (EPIs) among the top 1000 K562 EPIs. (G) Hierarchical clustering of ALL (Orange) and normal (Cyan) samples based on the selected ALL EPIs (Red) and normal EPIs (Blue). The heatmap represents the model prediction probability. The larger the value, the redder it is. (H) Differential gene expression (DGE) analysis for the 62 ALL specific genes derived from 137 predicted ALL EPIs.

RepliChrom achieved strong performance across all six cell lines, with AUPRC values exceeding 0.82 (baseline = 0.05) and AUROC values above 0.94 (Figure 2A, Figures S4, S5A–I). A cell line‐based general model trained on pooled data further improved performance, reaching an AUPRC > 0.88 and AUROC > 0.95 (Figure 2A, Figures S4G–I, S5G–I). Cross‐sample evaluations revealed decreased accuracy across cell lines compared to within‐line predictions (Figures 2B, S5J), reflecting the cell line‐specific nature of RT signals. Notably, the cell line‐based general model effectively mitigated this variability (Figures 2B, S5J), supporting its applicability in broader contexts.

To validate the reliability of RepliChrom, we first compared the RF model with other algorithms, including XGBoost, AdaBoost, Decision Tree, Gradient Boosting, Convolutional Neural Network, Long Short‐Term Memory, and Transformer. RF achieved the best performance (Figure S6A), likely due to the relatively simple and low‐complexity nature of RT features. Second, we compared RepliChrom with classic EPI predictors such as TargetFinder, JEME, RIPPLE, and Lollipop. RepliChrom outperformed most methods and achieved performance comparable to Lollipop, despite using only RT data instead of multiple epigenetic inputs (Figure S6B–F). Finally, RepliChrom trained on distance alone are mostly hovered around baseline, indicating that distance is properly controlled between positive and negative samples (Figure S6G). These results highlight the efficiency and robustness of RepliChrom.

Next, we analyzed 482 RT features to identify key predictors of EPIs. Feature importance analysis showed that promoter‐derived signals contributed more than enhancer‐derived ones (Figure S7A). Using incremental feature selection, we found that the top 30 promoter‐RT features achieved optimal performance (Figures S7B, S8). Models trained with promoter‐RT features alone outperformed those using enhancer features (Figure S7C–F). Additionally, 500 bp windows provided the most informative signals, while larger windows introduced noise, especially in the general model (Figure S7G–I). These results highlight the importance of fine‐resolution promoter RT features in EPIs prediction.

### RepliChrom generalizes across multiple chromatin interaction technologies

To evaluate the generalizability of RepliChrom, we applied it to ChIA‐PET and the highly sensitive transposase‐mediated analysis of chromatin (Hi‐TrAC) datasets from the GM12878 cell line [15, 16]. RT signals showed strong concordance with POLR2A/CTCF ChIA‐PET and Hi‐TrAC loops across chromosome 2 (Figure S9A), and RepliChrom successfully predicted over 60% of these independent loop interactions (Figure 2C). High‐confidence predictions were enriched for active histone marks such as H3K27ac and H3K4me3, indicating biological relevance (Figure S9B).

We further trained EPIs prediction models using RT‐encoded ChIA‐PET and Hi‐TrAC datasets within the RepliChrom framework (Figure S10, Table S2). These models achieved high AUPRC and AUROC scores across datasets (Figures S9C–G, S11). Among them, the POLR2A ChIA‐PET model achieved the highest loop prediction accuracy (>75%) in cross‐data set test (Figure S9H). These findings demonstrate that RepliChrom generalizes well to high‐resolution chromatin interaction data across different experimental platforms.

To further assess the robustness and cross‐platform applicability of RepliChrom, we applied the RT‐based EPIs prediction models trained on Hi‐C, ChIA‐PET, and Hi‐TrAC data to an independent Chromosome Conformation Capture Carbon Copy (5C) data set from the GM12878 cell line [17]. Notably, integrating predictions from all four models yielded the highest concordance with the 5C data, accurately recovering 81 out of 91 interactions (Figures 2D,E, S12), underscoring the complementary strengths and predictive accuracy of RepliChrom across different chromatin conformation capture technologies.

### RepliChrom reveals specific EPIs at key leukemia oncogenes

To explore the application of RepliChrom in cancer, we applied the K562‐trained model to RT data from acute lymphoblastic leukemia (ALL) and normal samples [18]. Using enhancer and promoter annotations from FANTOM and ENCODE, we generated over 5 million candidate EPIs, from which the top 1000 predictions were selected. RT profiles from ALL and normal samples were used to re‐encode these EPIs, enabling sample‐level prediction matrix construction (Figure S13A).

As a result, high‐confidence EPIs anchors were enriched for active histone marks and transcription factors such as H3K27ac, H3K4me3, CTCF, RAD21, and POLR2A, supporting their biological relevance (Figure S13B). From top 1000 K562 EPIs, we identified 137 ALL‐specific, 809 common, and 54 normal‐specific EPIs (Figures 2F, S13F, Table S3). Notably, known oncogenes such as *NPM1* were found among the ALL‐specific EPIs. Hierarchical clustering based on prediction scores of ALL‐EPIs and normal‐EPIs clearly separated ALL from normal samples (ARI = 1.00, CH = 110.66) (Figures 2G, S13G), indicating that RT‐based EPIs patterns can serve as discriminative features.

Further analysis revealed 36 ALL‐related genes overlapping with EPIs anchors, many of which (e.g., *NPM1*, *CD164*, *HDAC11*) are associated with poor prognosis in ALL or AML. Differential expression analysis identified 15 significantly upregulated genes in ALL (log2 fold change > 1 and *p* < 0.05), some of which are typical ALL pathogenesis‐related genes, such as *NPM1* [19] (Figure 2H). Next, gene ontology (GO) enrichment highlighted functions in nucleosome assembly and DNA packaging (Figures S13C, S13H). These results demonstrate the potential of RepliChrom to identify oncogenic 3D chromatin interactions from RT signals, offering insight into leukemia‐associated regulatory mechanisms.

We further tested the top 1000 K562 EPIs for their ability to distinguish B‐ALL and T‐ALL subtypes. Based on 31 T‐ALL and 71 B‐ALL subtype‐specific EPIs, hierarchical clustering showed limited separation (ARI = 0.18, CH = 13.29), suggesting conserved 3D chromatin interactions across ALL subtypes (Figure S13D,E).

## CONCLUSION

Taken together, we developed RepliChrom, an interpretable machine learning framework that leverages DNA RT to predict EPIs across diverse human cell lines. Our results show that RT is both biologically informative and computationally effective for modeling long‐range chromatin interactions. RepliChrom generalizes well across platforms (Hi‐C, Hi‐TrAC, ChIA‐PET, 5C) and cell types (K562, GM12878, HUVEC, NHEK, HeLaS3), and achieves performance comparable to or better than existing models like TargetFinder and RIPPLE, despite using only RT as input.

Clinically, we applied RepliChrom to ALL samples to identify cancer‐specific EPIs. Notably, several high‐confidence EPIs were able to distinguish ALL from normal samples. Among these, we identified the well‐known leukemia gene *NPM1* and other factors such as *CD164*, *HDAC11*, and *GLUL* as aberrantly expressed and associated with disease‐specific chromatin loops. GO enrichment analysis of genes linked to ALL‐specific EPIs revealed significant involvement in processes such as chromatin organization and megakaryocyte differentiation, reinforcing their potential roles in leukemogenesis. These findings suggest that RT‐associated EPIs could serve as a novel layer of epigenetic regulation in cancer and offer insights for biomarker discovery.

While our study highlights the predictive power of RT for 3D genome organization, several directions remain for future work. We will aim to address batch effects in Repli‐Seq data and extend RepliChrom to single‐cell and subtype‐specific contexts to capture regulatory heterogeneity. Emerging technologies like single‐cell Hi‐C and HiRES [20] offer opportunities to integrate RT with spatial‐temporal chromatin features at cellular resolution. Incorporating additional epigenetic marks may further improve model accuracy and interpretability (Figure S14). As more RT and chromatin interaction datasets become available, expanding the model to a broader range of cell types, tissues, and cancers will be essential for enhancing its generalizability.

## Supporting information


**Figure S1:** DNA replication timing (RT) relationship to 3D chromatin structure.
**Figure S2:** Analysis of replication timing (RT) profiles across different cell types and conditions.
**Figure S3:** Hi‐C training dataset construction and replication timing (RT) features extraction.
**Figure S4:** The predictive performance (AUPRC) of RepliChrom across different cell lines.
**Figure S5:** The predictive performance (AUROC) of RepliChrom across different cell lines.
**Figure S6:** RepliChrom compared with other models.
**Figure S7:** Feature importance analysis of RepliChrom.
**Figure S8:** Importance scores of replication timing (RT) features in promoters and enhancers.
**Figure S9:** RepliChrom demonstrates strong cross‐platform generalization in predicting chromatin loops.
**Figure S10:** Compare the data of Hi‐C, ChIA‐PET and Hi‐TrAC.
**Figure S11:** Evaluation of the generalization ability of RepliChrom in ChIA‐PET and Hi‐TrAC datasets.
**Figure S12:** Validation of RepliChrom predictions using 5C chromatin interaction data.
**Figure S13:** Model application in acute lymphoblastic leukemia (ALL) samples.
**Figure S14:** Compare replication timing (RT) feature with epigenetics signals.

**Table S1:** Positive and negative of Hi‐C datasets.
**Table S2:** Positive and negative of Hi‐TrAC and ChIA‐PET datasets.
**Table S3:** The acute lymphoblastic leukemia (ALL) enhancer‐promoter interactions (EPIs), common EPIs, and normal EPIs among the predicted K562 top 1000 EPIs.

## Data Availability

Source code of RepliChrom can be accessed at: https://github.com/DaoFuying/RepliChrom. Supplementary materials (methods, figures, tables, graphical abstract, slides, videos, Chinese translated version and update materials) may be found in the online DOI or iMeta Science http://www.imeta.science/. The data that support the findings of this study are openly available in Zenodo at https://zenodo.org/records/11398547.
