# Supplementary material for: RepliChrom: Interpretable machine learning predicts cancer‐associated enhancer‐promoter interactions using DNA replication timing
Source: Imeta. 2025 May 27;4(4):e70052. doi: 10.1002/imt2.70052 (PMC12371266; doi:10.1002/imt2.70052)
Supplement: Supplementary file 1 — Figure S1: DNA replication timing (RT) relationship to 3D chromatin structure. Figure S2: Analysis of replication timing (RT) profiles across different cell types and conditions. Figure S3: Hi‐C training dataset construction and replication timing (RT) features extraction. Figure S4: The predictive performance (AUPRC) of RepliChrom across different cell lines. Figure S5: The predictive performance (AUROC) of RepliChrom across different cell lines. Figure S6: RepliChrom compared with other models. Figure S7: Feature importance analysis of RepliChrom. Figure S8: Importance scores of replication timing (RT) features in promoters and enhancers. Figure S9: RepliChrom demonstrates strong cross‐platform generalization in predicting chromatin loops. Figure S10: Compare the data of Hi‐C, ChIA‐PET and Hi‐TrAC. Figure S11: Evaluation of the generalization ability of RepliChrom in ChIA‐PET and Hi‐TrAC datasets. Figure S12: Validation of RepliChrom predictions using 5C chromatin interaction data. Figure S13: Model application in acute lymphoblastic leukemia (ALL) samples. Figure S14: Compare replication timing (RT) feature with epigenetics signals. [file IMT2-4-e70052-s001.docx]

**Supporting information to**

**RepliChrom: Interpretable machine learning predicts cancer-associated enhancer-promoter interactions using DNA replication timing**

**Running title:** DNA replication timing predicts cancer 3D chromatin interactions

Fuying Dao^1,2^, Benjamin Lebeau^2^, Crystal Chia Yin Ling^2^, Mi Yang^1^, Xueqin Xie^1^, Melissa Jane Fullwood^2, 3, *^, Hao Lin^1, *^, Hao Lyu^1, *^

^1^Department of Clinical Laboratory, Sichuan Clinical Research Center for Cancer, Sichuan Cancer Hospital & Institute, Sichuan Cancer Center, School of Life Science and Technology, University of Electronic Science and Technology of China, Chengdu, 610054, China

^2^School of Biological Sciences, Nanyang Technological University, Singapore, 639798, Singapore

^3^Institute of Molecular and Cell Biology, Agency for Science, Technology and Research (A*STAR), Singapore, 138673, Singapore

^*^Correspondence: [mfullwood@ntu.edu.sg](mailto:mfullwood@ntu.edu.sg) (Melissa Jane Fullwood), [hlin@uestc.edu.cn](mailto:hlin@uestc.edu.cn) (Hao Lin), [hao.lyu@uestc.edu.cn](mailto:hao.lyu@uestc.edu.cn) (Hao Lyu)

## Supplementary methods

### Replication timing data collection and processing

The replication timing (RT) data used in this study were obtained from the laboratory of Dr. David M. Gilbert, including both the RT profiles of cell lines used for model training (e.g., K562, GM12878, HUVEC, NHEK, HeLaS3) [1] and those of acute lymphoblastic leukemia (ALL) and matched normal samples [2]. These datasets were generated using the Repli-Seq protocol as described in Hansen et al. [3], which combines BrdU labeling of newly synthesized DNA with fluorescence-activated cell sorting (FACS) of early and late S-phase fractions, followed by high-throughput sequencing. RT signals were calculated as the log2 ratio of sequence read counts between early and late S-phase fractions, providing genome-wide RT profiles at ~50 Kb resolution. All data were preprocessed using a uniform pipeline to ensure consistency across samples and minimize technical variability.

### Replication timing visualization

To explore the RT characteristics of different cell lines, we utilized the Nucleosome Browser tool [4] to visualize the RT profiles of biological replicates across five cell lines on chromosome 2 (**Figure S2A**). We observed significant variations in the distribution of RT signals among different cell types, indicating heterogeneity in RT profiles. Simultaneously, the same cell types exhibit highly conserved RT profiles among biological replicates, such as chronic myelogenous leukemia cells (K562), cervical cancer cells (HeLaS3), and lymphoblastoid cells (GM12878). This suggests a tendency towards stability in the RT profiles among cells of the same type.

To further investigate the stability of RT profiles within a specific cell type, we analyzed four established nonleukemic EBV-transformed mature human B lymphoblastoid cell lines: C0202, NC-NC, GM06990, and GM06999. The **Figure S2B** displays the RT profiles of these four nonleukemic B lymphoblastes from different individuals over an exemplary 25 Mb chromosomal segment, with different colors representing cells from different individuals. We found that RT features are stable characteristics of mature human B cells, supporting the conclusion that RT profiles exhibit robust stability among common cell types. Moreover, based on the conservation of RT within the same cell type and the specificity of RT across different cell types, we can infer that cell identity may be dictated by specific RT signals.

Next, we evaluated the differences in RT between normal samples and ALL samples. As shown in **Figure S2C**, the RT profiles of non-leukemic mature B lymphocytes display similar signal distributions, while the RT profiles of B-ALL patients show greater individual variability. Compared to normal B cells, we observed more pronounced replication abnormalities in the region of 55-60 Mb in four B-ALL patient samples, where the replication status shifted from early to late. Furthermore, the intensity of replication signals in B-ALL patient samples diverged, showing a range of [-2, 2] compared to normal B cells, which ranged from [-1, 1]. Then, we performed unsupervised hierarchical clustering using RT information from the 55-60 Mb region of chromosome 2 for both ALL and normal samples. As illustrated in **Figure S2D**, Fragmented RT information can distinguish ALL samples from normal samples (Adjusted Rand Index (ARI) = 1.00, Calinski-Harabasz Index (CH) = 93.78). Therefore, the replication program in ALL significantly differs from that in non-leukemic normal cells.

To gain new insights into the RT differences among various ALL subtypes, we mapped the RT signals of the T-ALL subtype sample (10-828) alongside the B-ALL subtype samples 10-822 and 10-838 on a segment of chromosome 12 (**Figure S2E**). Interestingly, all three ALL samples exhibited a sharp transition to late replication status in the 12.95 Mb region, reflecting a certain degree of stability in the replication program across different ALL subtypes. This region is located within the tumor suppressor gene *GPRC5A* [5], suggesting that mutations in *GPRC5A* leading to sustained activation of *STAT3* may be a pathological factor in these ALL patients [6]. Furthermore, B-ALL subtype samples 10-822 and 10-838 were from patients who experienced relapsed disease and subsequently died. Therefore, further investigation is warranted to determine whether *GPRC5A* could serve as a potential prognostic marker. Inspired by this, we validated the ability of RT signals to distinguish between ALL subtypes based on RT information in the 55-60 Mb region of chromosome 12. As shown in **Figure S2F**, fragmented RT information was less effective at identifying of ALL subtype’s samples (ARI = -0.04, CH = 33.25).

To counteract the negative impact of heterogeneity among ALL subtypes on clustering performance, we endeavored to find more informative features to bridge the gap left by the direct characterization of ALL and normal samples using RT signals. Over the past decade, significant efforts have been made to deciphering the positive correlation between the DNA replication program and 3D genomic structure [7]. Solovei et al. have proved the spatial organization of late-replicating chromatin at the nuclear periphery and around the nucleolus, which early replicating chromatin is found in more central regions of the nucleus [8]. As shown in **Figure S2G**, we illustrated the A/ B compartments and corresponding RT signal of GM12878 and IMR90 at regions of 150-249.3 Mb in chromosome 1, where early replication region corresponds to A compartments and the late replication region corresponds to B compartments. Therefore, RT information holds great potential in predicting the spatial organization of genome. Drawing on this innovative regulatory mechanism, we plotted the distribution of RT signals across peaks of chromatin interaction intensity in the 3D genome of chromosome 2 in the K562 cell line. As depicted in **Figure S2H**, there is a strong correlation between the peaks and troughs of RT and Hi-C signals, highlighting the value of RT signals in representing enhancer-promoter interactions (EPIs), thereby facilitating de novo prediction of chromatin interactions in cancer samples.

### Datasets collection and processing for Hi-C, ChIA-PET, Hi-TrAC, and 5C

Raw Hi-C data were downloaded from Gene Expression Omnibus (GEO) with accession number GSE63525 [9]. To simplify the analysis, adjacent enhancers or promoters within 500 bp of each other are considered a single enhancer or promoter. Positive EPIs are formed by pairing the combined enhancers and promoters (**Figure S3A**).

To generate positive and negative datasets for training RepliChrom models, we defined enhancers for five widely studied cell lines (K562, GM12878, NHEK, HUVEC, and HeLaS3), we collected 32,693 strictly annotated enhancers from the FANTOM database [10]. Additionally, strong and weak enhancers were obtained from the UCSC database [11], annotated using the ChromHMM tool [12]. Active enhancers were identified by overlapping enhancers with H3K27ac signal peaks from ENCODE H3K27ac ChIP-seq data [13]. Promoters were defined as genomic regions spanning 1,000 bp upstream to 100 bp downstream of transcription start sites (TSS), using the V19 TSS annotation from ENCODE [14]. Promoters were considered "active" if their corresponding gene expression exceeded 0.3 reads per kilobase per million mapped reads (RPKM) in RNA-Seq data, with an irreproducibility discovery rate (IDR) set to 0.1. For cell lines lacking RNA-seq data, all promoters were treated as active. Using Hi-C interaction matrices and significant chromatin loop files from the GEO database [9], enhancer-promoter pairs overlapping with loop anchors were designated as positive EPIs, provided the enhancer and promoter were at least 500 bp apart. Non-interacting pairs (non-EPIs) were defined as enhancer-promoter pairs located within 5 Kb to 2 Mb that did not overlap with any Hi-C loop anchors. To ensure balanced datasets, we generated samples with a 1:20 ratio of positive to negative pairs, matching the distance distributions between EPIs and non-EPIs (**Figure S3C-H**). Detailed information on Hi-C data is presented in the **Table S1**.

ChIA-PET can capture genome-wide chromatin interactions mediated by a specific DNA-associated protein [15]. And Hi-TrAC technology was recently developed to map chromatin loops among transcription and chromatin regulator elements in accessible genomic regions [16]. Both techniques offer high resolution chromatin interaction data compared to Hi-C.

Raw Hi-TrAC and ChIA-PET data were downloaded from GEO with accession number GSE180175 [16] and GSE72816 [17], respectively. The anchor lengths of Hi-TrAC and ChIA-PET are shorter than those of Hi-C anchors (**Figure S10A**). In addition, Hi-TrAC and ChIA-PET can generate more anchors (**Figure S10B**). This means that there is sufficient data for each anchor to overlap with only one enhancer or promoter. Consequently, one-to-one EPI is regarded as positive EPI (**Figure S10C**), providing a more rigorous positive dataset. For the generation of negative EPIs, enhancers or promoters that did not overlap with positive anchors were selected, and the same distance-distribution dataset was formed in a ratio of 1:20 to positive EPIs (**Figure S10D-F**). Detailed information about Hi-TrAC and ChIA-PET datasets is provided in the **Table S2**.

To evaluate the quality of the computational interaction maps generated by RepliChrom, we compared the EPIs predicted by the Hi-C model, Hi-TrAC model, and POLR2A/ CTCF ChIA-PET model with Chromosome Conformation Capture Carbon Copy (5C) data (GSE39510) [18].

### Multi-scale replication timing feature extraction strategy

To extract more information from RT profile, anchors are redefined by extending the area around the midpoint of each anchor (30 Kb upstream and downstream). As shown in Equation (1), the RT can serve as a feature of each anchor, which is obtained from the combination of features from windows of 7 different lengths (0.5 to 7.5 Kb, with a step size of 1 Kb). An anchor of length *L* bp is divided into *n* segments using windows of length *winL* bp. If a window overlaps with a RT segment, it is encoded with the corresponding RT value; if not, it is encoded with 0. For each window, each anchor can generate a *bn*-dimensional feature vector. Ultimately, the RT feature vector of each anchor is obtained by combining the features from the 7 windows. The final feature set can be formed by concatenating the feature subsets of the left and right anchors of EPI.

$\left\{ \begin{aligned} RT=\left[ \mathrm{RT}_{1}\mathrm{RT}_{2} \cdots\mathrm{RT}_{b} \right] \\ \mathrm{RT}_{b}=\left[ \mathrm{RT}_{b1} \mathrm{RT}_{b2} \cdots\mathrm{RT}_{\mathrm{bn}} \right], n=\frac{L}{\mathrm{winL}} \\ \mathrm{RT}_{\mathrm{bn}}=\frac{\sum_{i=0}^{k} \mathrm{RT}_{i}}{k} \end{aligned} \right.$ (1)

The schematic diagram for RT feature extraction based on a specified window size can be found in **Figure S3B**. For example, with a window size of 500 bp and a sliding step size of 500 bp, an enhancer or promoter region of 60 Kb will yield 120 (60,000/ 500) RT features. By integrating the RT features of both enhancer and promoter regions, a total of 240 (120×2) dimensions of RT features are generated. Following this approach, the RT features obtained from different window sizes are ultimately combined into a final RT feature set comprising 482 dimensions.

### RepliChrom model training

The random forest (RF) algorithm is a flexible and practical machine learning method based on Bagging. It consists of a large number of individual decision trees that operate as an ensemble [19]. In this study, we used an R package ranger (https://cran.r-project.org/web/packages/ranger) to implement RF classifiers using the default package parameters, specifically with 500 trees. 5-fold cross validation and multiple random training were used to evaluate model performance. For the latter, we performed 100 iterations trainings, each using 8/10 of the data as the training set and the remaining data as the test set.

All models were trained and evaluated on a high-performance Linux server equipped with an NVIDIA Tesla P100 GPU (12GB memory), dual Intel Xeon E5-2698 v4 CPUs (2.2GHz, 40 cores total), and 512 GB of RAM. The training process was implemented in Python 3.9 and R 4.3.1. For a typical cell line–specific model (e.g., K562), the complete process including RT feature extraction, 5-fold cross-validation, and 100 rounds of random training took approximately 50 minutes. Prediction tasks for the whole genome potential EPIs predictions typically completed within 20 minutes using CPU resources alone, without requiring GPU acceleration.

### RepliChrom model compares with other methods

To assess the reliability of the algorithm implemented in RepliChrom, we first compared the performance of the RF algorithm with other machine learning and deep learning approaches, including XGBoost, AdaBoost, Decision Tree, Convolutional Neural Network (CNN), Long Short-Term Memory (LSTM), and Transformer. Our results indicate that compared to deep learning, general machine learning methods are more suitable for the feature patterns of RT, with RF yielding superior results (**Figure S6A**). We speculate that the RT signal may exhibit lower complexity or fewer non-linear relationships, making it easier for general models like RF to effectively capture the underlying patterns without overfitting. In contrast, deep learning models, which are well-suited for complex non-linear relationships, may be prone to overfitting when simpler relationships are adequate.

Second, we evaluated RepliChrom against classic models, including TargetFinder [20], JEME [21], RIPPLE [22], and Lollipop [23]. While TargetFinder and JEME have limitations, such as high data overlap [24], they still serve as valuable benchmarks. As shown in **Figure S6B-D**, RepliChrom outperformed TargetFinder, JEME, and RIPPLE in both K562 and GM2878 cell lines, highlighting the utility of RT in predicting EPIs. Although Lollipop achieved better results (**Figure S6E**), it is worth noting that Lollipop requires 15 epigenetic modification signals as input, whereas RepliChrom achieves comparable performance using only the RT profile (**Figure** **S6F**). This demonstrates the efficiency of RepliChrom in EPI prediction with minimal input data, offering a novel and effective approach.

### Replication timing feature selection

To decipher the determinants of EPIs, we calculated the importance score for each feature using RF, where a higher score indicates greater contribution to the model. The features were ranked from largest to smallest according to their importance scores. And 482 feature subsets were constructed on the basis of the incremental feature selection (IFS) rule [25].

### RepliChrom application in acute lymphoblastic leukemia (ALL) samples

To explore the ability of specific 3D chromatin interaction information to differentiate between ALL samples and normal samples, we utilized the K562 model to identify significant EPIs across the genome as follows (**Figure S13A**). At first, we downloaded RT data for ALL samples and normal samples from the GEO database (GSE37987) [2]. Secondly, human enhancers were collected from the FANTOM database [26], and promoters were derived from genome-wide transcription start site information from the ENCODE database [14]. By setting the distance between enhancers and promoters to 5 Kb-2 Mb, we generated a total of 5,375,351 potential K562 EPIs were generated. Thirdly, all potential EPIs predicted by K562 model were ranked by their prediction probabilities, with the top 1,000 considered significant EPIs. Fourthly, we encoded these significant EPIs based on the RT information of ALL samples and normal samples, and inputted them into the RepliChrom model. Thus, we obtained a matrix composed of prediction probabilities, where the rows represent EPIs, and the columns represent ALL samples or normal samples. Finally, we analyzed the matrix to identify significant EPIs that could distinguish ALL samples from normal samples.

To evaluate the reliability and biological relevance of our prediction results, we analyzed EPI anchors in K562 cells by stratifying them based on predicted probabilities. As shown in **Figure S13B**, EPI anchors with high prediction probabilities were significantly enriched for active histone modifications (e.g., H3K27ac, H3K4me3) and transcriptional regulators, including CTCF, RAD21, and POLR2A. In contrast, low-confidence EPI regions showed minimal enrichment for these features. These observations indicate that RT signals captured by RepliChrom are predictive of transcriptionally active and structurally organized genomic regions, and further support a mechanistic association between replication programs, chromatin accessibility, and transcription factor occupancy.

Next, we selected 137 ALL EPIs, 809 common EPIs, and 54 normal EPIs from the top 1,000 K562 EPIs based on prediction probability (**Figure 2F, Table S3**). We found the oncogene *NPM1* [27], a well-known pathogenic gene associated with ALL, among the ALL EPIs. Subsequently, hierarchical clustering was performed on ALL and normal samples based on the model-predicted probabilities of ALL EPIs and normal EPIs (**Figure 2G**). The clustering results showed that the ALL and normal-specific EPIs recognized by RepliChrom were able to correctly distinguish between ALL samples and normal samples (ARI = 1.00, CH = 110.66). This indicates that RT signal can reveal 3D chromatin interaction information with biomarker characteristics.

Furthermore, when 137 ALL EPIs were mapped onto the hg19 genome, 36 genes were found to overlap with the promoters of these loops. Notably, genes such as *NPM1*, *CD164*, *HDAC11*, and *GLUL*, have all been associated with poor prognosis in cases of ALL or acute myeloid leukemia (AML). To further identify global gene signatures intrinsic to ALL based on these 36 ALL genes, we first performed a differential gene expression analysis by comparing the expression levels of all 36 genes in ALL samples versus normal samples from the TCGA database [28] (**Figure 2H**). This analysis identified a subset of 15 genes that were significantly overexpressed in ALL (log2 fold change > 1 and *p* < 0.05), some of which are typical ALL pathogenesis-related genes, such as *NPM1* [27]. Next, we conducted a gene ontology (GO) analysis of the 36 key genes. As shown in **Figure S13C**, they are mainly enriched in biological processes such as nucleosome assembly and DNA packaging, and they are primarily involved in cellular components including nucleosome, DNA packaging complex, and protein-DNA complex. Therefore, RepliChrom was able to predict potentially oncogenic chromatin interactions that drive ALL or AML, using available RT signals.

Similarly, we investigated the ability of the top 1,000 K562 EPIs to distinguish between B-ALL and T-ALL subtype samples. As shown in **Figure S13D-E**, T-ALL and B-ALL samples were clustered based on the predicted probabilities of 31 T-ALL EPIs and 71 B-ALL EPIs obtained by the model. We found that the effect of hierarchical clustering was suboptimal (ARI = 0.18, CH = 13.29), indicating that 3D chromatin interaction information is conserved among ALL subtypes samples.

Following the same pipeline, we applied the cell line-based general model to predict all potential genome-wide EPIs and selected the top 1,000 EPIs based on prediction probabilities. As shown in **Figure S13F**, these EPIs were classified into 211 ALL-specific, 88 normal-specific, and 701 common EPIs. Hierarchical clustering using the prediction probabilities of the 211 ALL-specific EPIs moderately separated ALL from normal samples (**Figure S13G**), though the performance was not as strong as that of the K562 model. GO enrichment analysis of the genes involved in ALL-specific EPIs revealed significant enrichment in pathways such as nucleosome assembly, protein-DNA complex assembly, and megakaryocyte differentiation (**Figure S13H**), suggesting strong biological relevance to leukemia.

## Supplementary figures


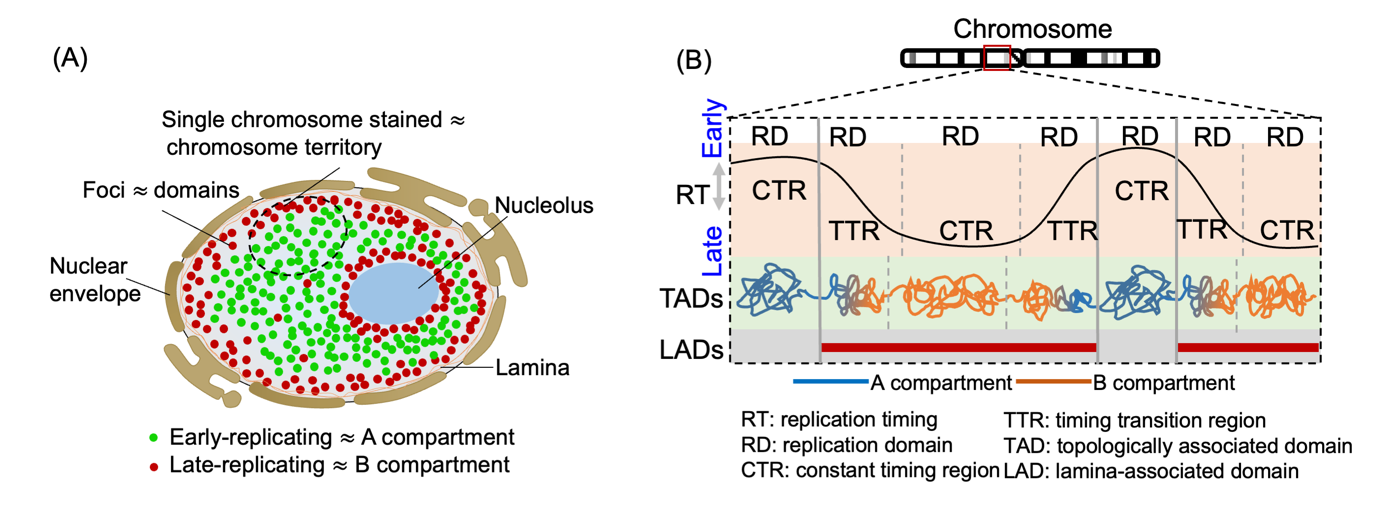


### Figure S1. Replication timing (RT) relationship to 3D chromatin structure. (A) RT illuminates genome architecture: nuclei after an early S pulse label (green) followed by several hours of a chase period and then a late S pulse label (red). In this model, observable foci of DNA synthesis correspond to the replication domains and early/ late-replicating chromatin corresponds to A/ B compartments [12]. (B) Current model of the relationship between RT and chromatin structure: Constant timing regions (CTRs), typically 1- 5 Mb in size, are divided by timing transition regions (TTRs). Each CTR contains one or more replication domains (RDs), which switch RT coordinately during cell fate changes and largely align with a subset of topologically associated domains (TADs), especially at compartment boundaries. Early CTRs align with A compartments, late CTRs with B compartments, and TTRs mark transitions between them. Both late CTRs and TTRs are often associated with lamina-associated domains (LADs).


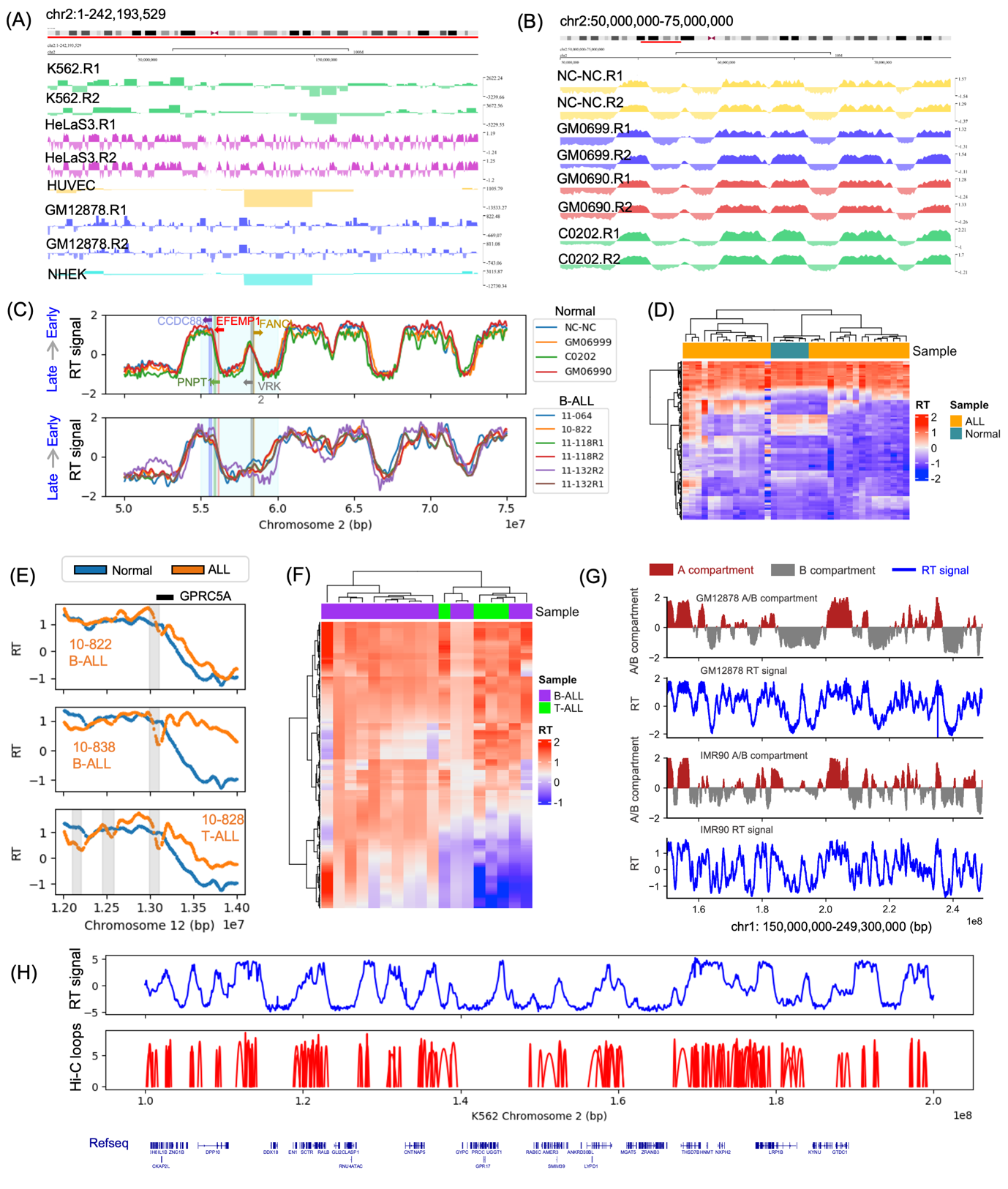


### Figure S2. Analysis of replication timing (RT) profiles across different cell types and conditions. (A) Visualization of RT profiles on chromosome 2 in five cell lines using the Nucleosome Browser tool. (B) RT profiles of four non-leukemic B lymphoblastoid cell lines (C0202, NC-NC, GM06990, GM06999) over a 25-Mb segment of chromosome 2. (C) Comparison of RT profiles between normal mature B lymphocytes and B-ALL patient samples. (D) Unsupervised hierarchical clustering ALL and normal samples based on RT information from the 55-60 Mb region on chromosome 2. (E) Mapping of RT signals in T-ALL and B-ALL subtype samples on chromosome 12: 12-14 Mb. (F) Unsupervised hierarchical clustering T-ALL and B-ALL subtype samples based on RT information from the 12-14Mb region on chromosome 12. (G) A/B compartments and RT signals on chr1: 150-249.3 Mb in GM12878 and IMR90 cell lines. (H) Correlation of RT and Hi-C signals in the K562 cell line on chromosome 2, demonstrating the alignment of RT with peaks of chromatin interaction intensity.


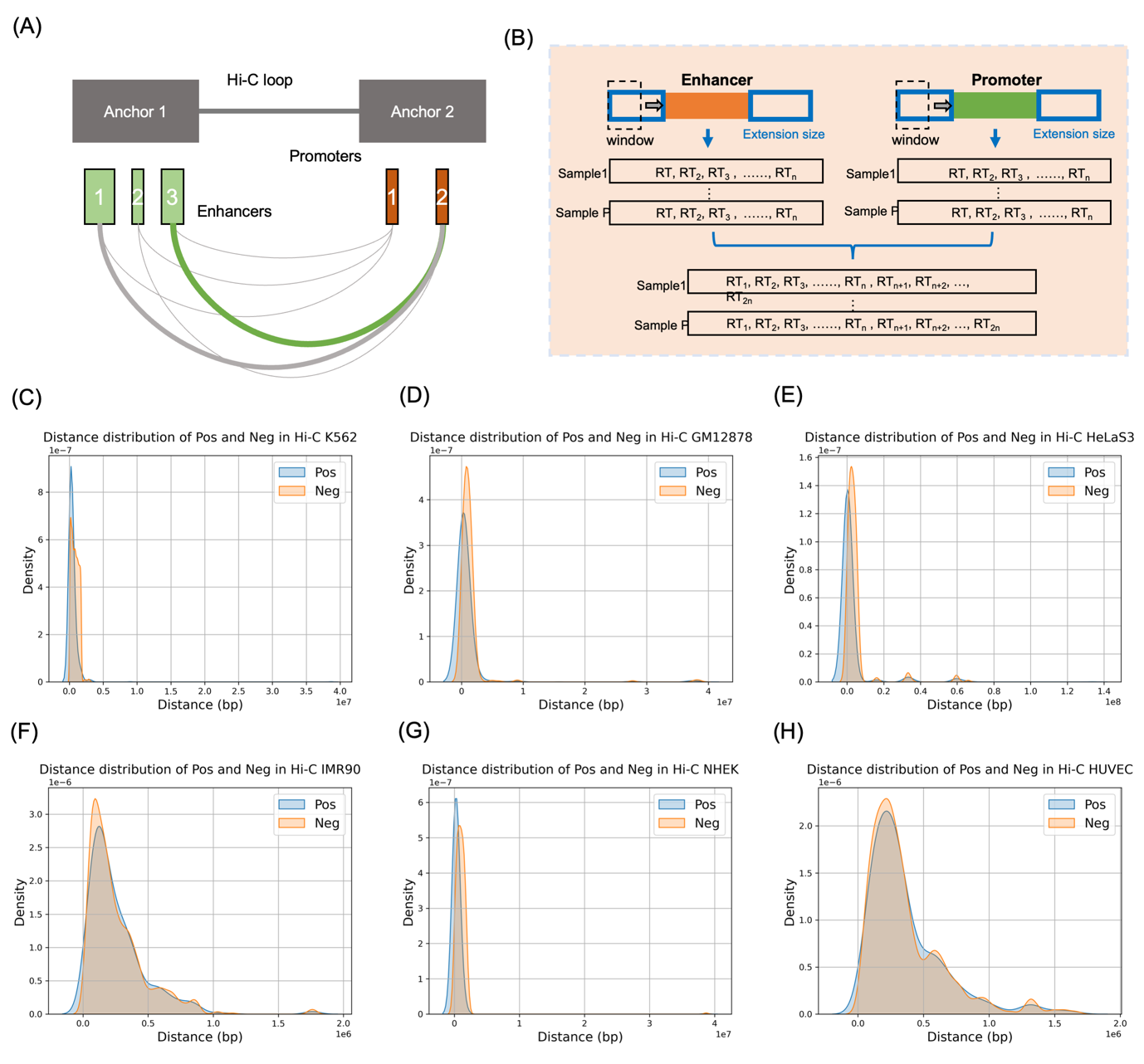


### Figure S3. Hi-C training dataset construction and replication timing (RT) feature extraction. (A) The positive EPIs extracted from Hi-C loop anchor. (B) Multi-scale RT feature extraction strategy. When extracting RT features using a fixed-size sliding window, each window corresponds to one RT value, and as the window slides, it generates (L/winL) RT feature values. By integrating the RT features of enhancers and promoters, a feature subset for that window size is formed. The final RT feature set is obtained by combining the feature subsets from different window sizes. L is the length of anchor, winL is the length of window. (C-H) The distance distribution of positive and negative set in Hi-C datasets of K562, GM12878, HeLaS3, IMR90, NHEK, and HUVEC cell lines.


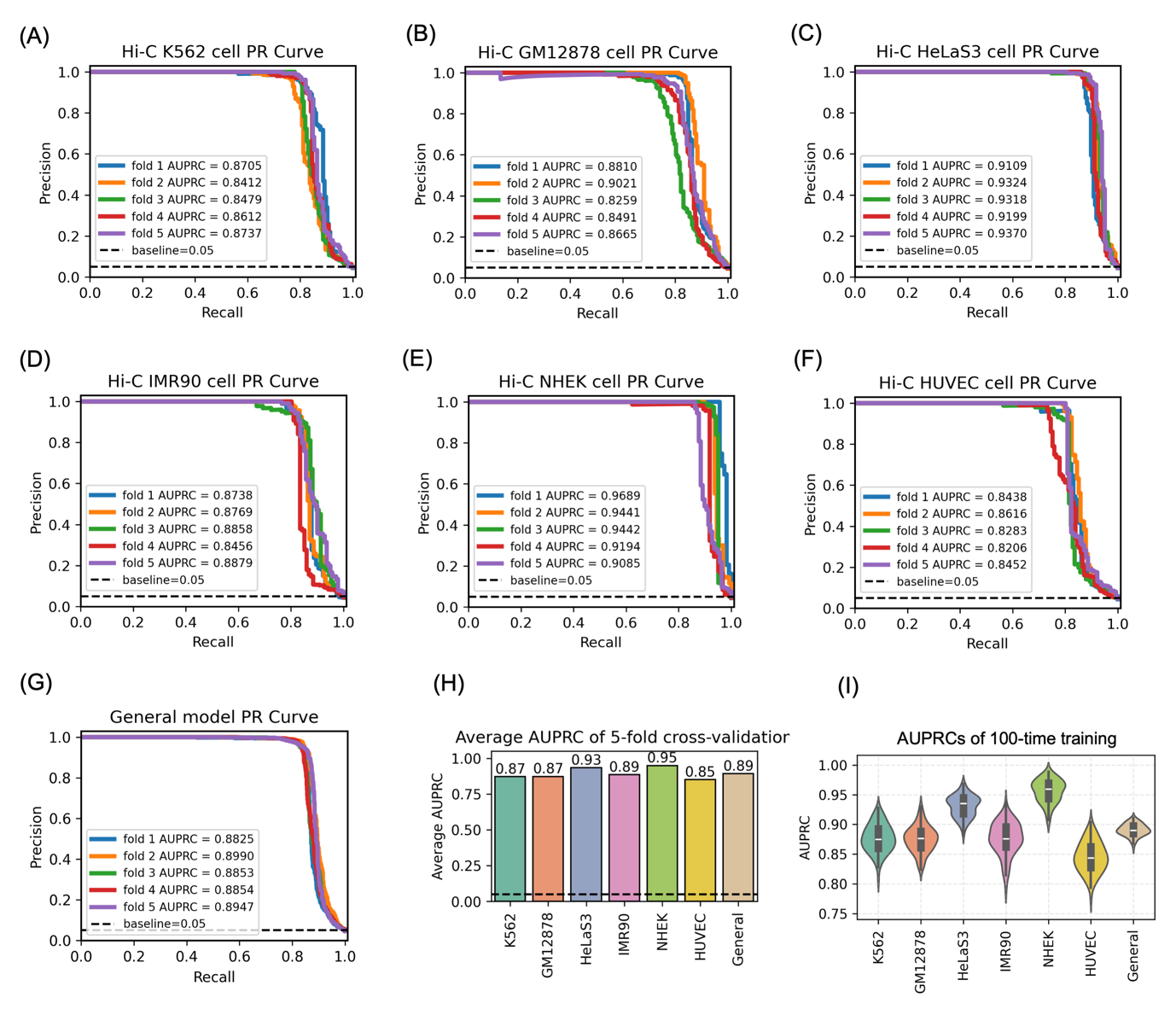


### Figure S4. The predictive performance (AUPRC) of RepliChrom across different cell lines. (A-G) Precision-Recall (PR) curves generated by RepliChrom for the K562, GM12878, HeLaS3, IMR90, NHEK, and HUVEC cell lines, as well as general model, are depicted. Each figure illustrates the results of 5-fold cross-validation, with each PR curve corresponding to one fold. The AUPRC serves as an effective metric for assessing model performance, with higher values indicating better performance. (H) The average AUPRC values produced by RepliChrom based on 5-fold cross validation across the six cell lines and General model. (I) The AUPRCs produced by RepliChrom based on 100 iterations model training and testing across the six cell lines and General model. AUPRC: Area Under the Precision-Recall Curve.


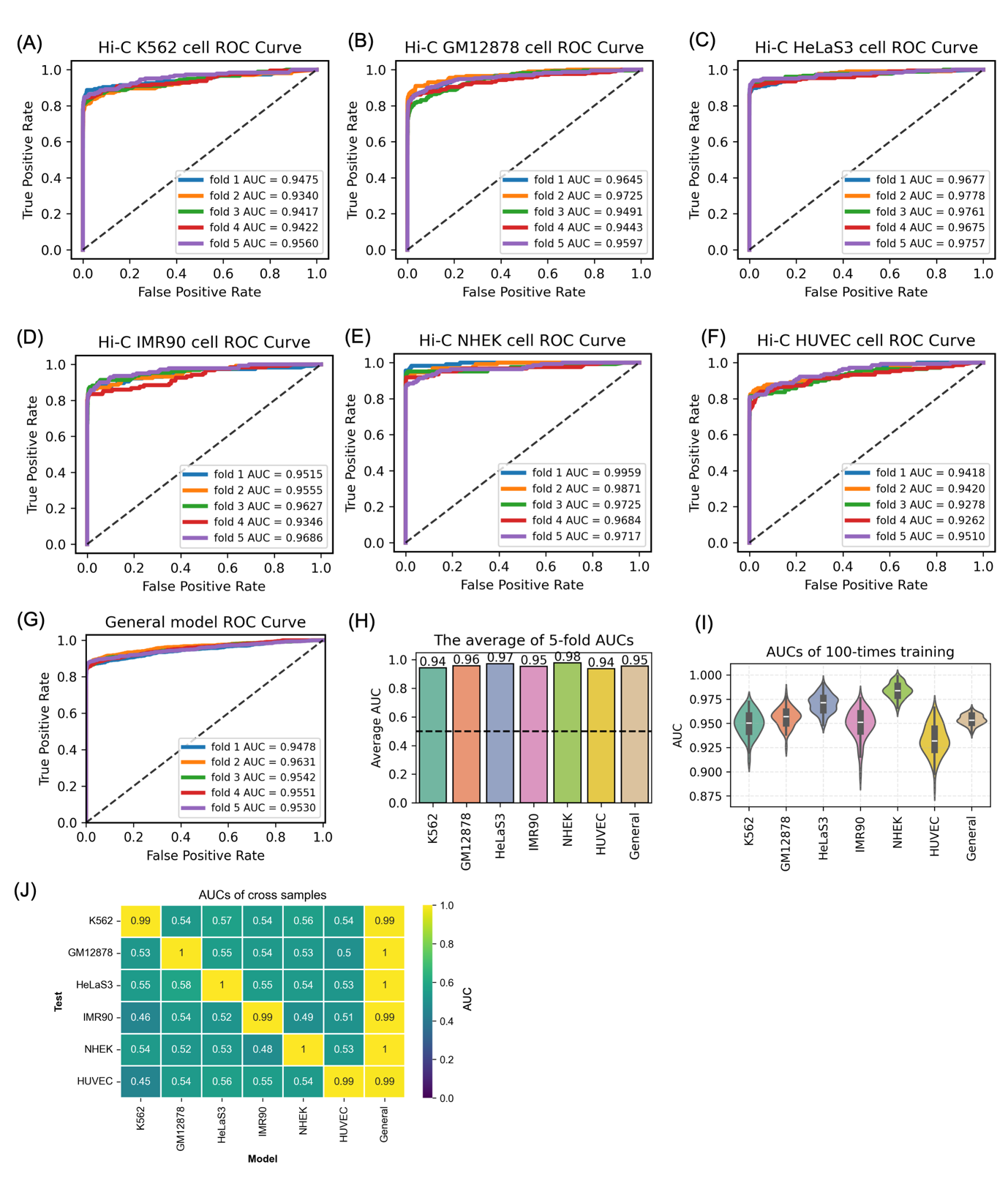


### Figure S5. The predictive performance (AUROC) of RepliChrom across different cell lines. The ROC curves generated by RepliChrom for the (A) K562, (B) GM12878, (C) HeLaS3, (D) IMR90, (E) NHEK, (F) HUVEC cell lines and (G) general model are depicted. Each figure illustrates the results of 5-fold cross-validation, with each ROC curve corresponding to each fold. The AUC serves as an effective metric for assessing model performance, with higher values indicating better performance. (H) The mean AUC generated by RepliChrom based on 5-fold cross-validation across the six cell lines. (I) The AUCs produced by RepliChrom based on 100 times of model training and testing across the six cell lines. (J) AUCs of cross-cell line based on RT information. AUROC: Area Under the Receiver Operating Characteristic.


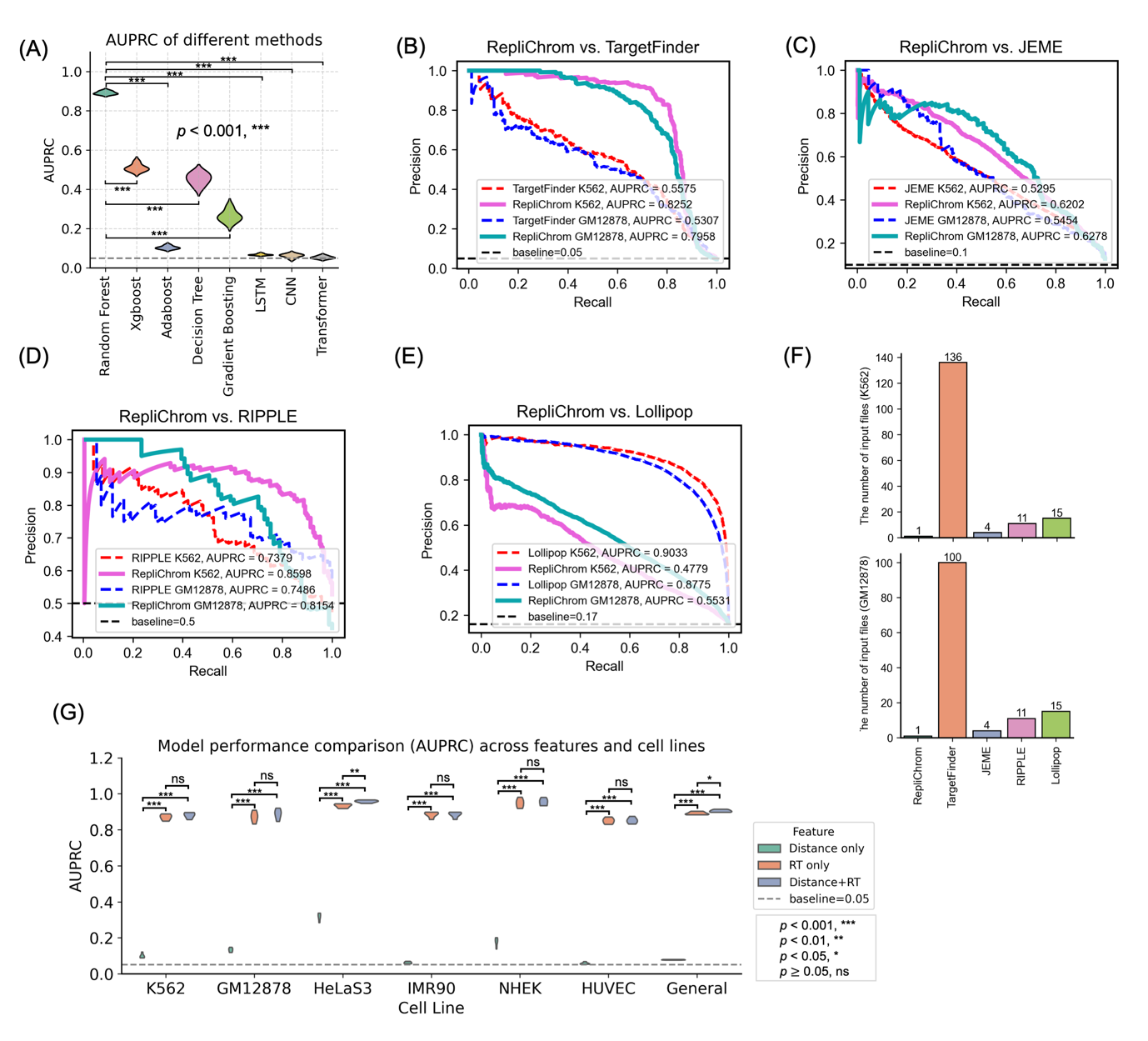


### Figure S6. RepliChrom compared with other models. (A) A comparison of Random Forest with other machine learning and deep learning methods is presented. (B-E) Precision-Recall curves for K562 and GM12878 cell lines when RepliChrom is compared with TargetFinder, JEME, RIPPLE, and Lollipop. (F) Number of input files for the model when using RepliChrom and other methods. (G) AUPRCs of models trained by using features based on distance only, replication timing (RT) only, and distance and RT were shown. AUPRC: Area Under the Precision-Recall Curve.


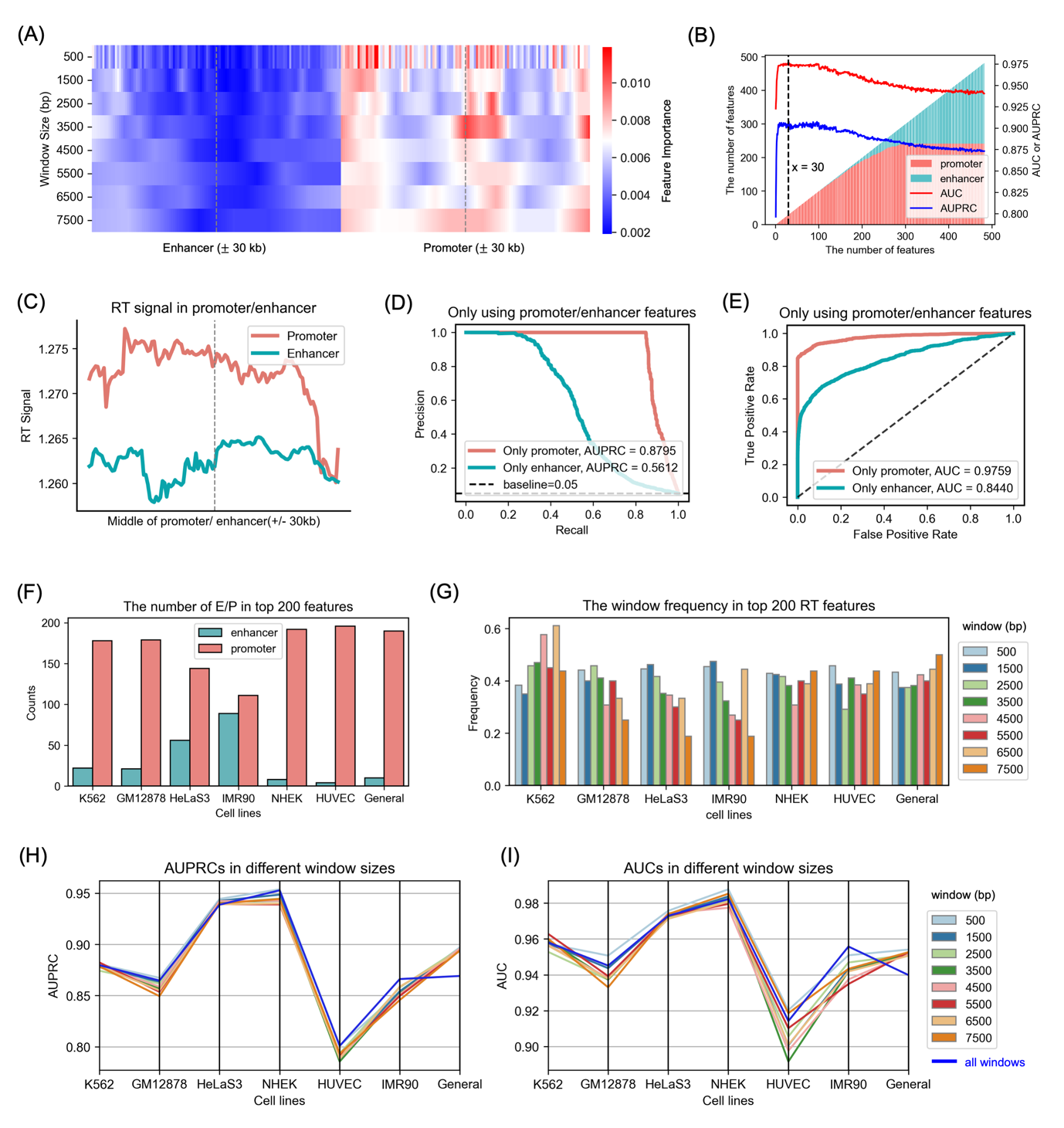


### Figure S7. Feature importance analysis of RepliChrom. (A) A heatmap to show the important score of RT feature in enhancer and promoter in general model, in which, the higher the score, the redder the color, indicating greater the contribution of the feature to the model. (B) Importance scores of RT signals in promoters and enhancers in general model. Red and blue lines represent AUC and AUPRC evaluation metrics, respectively. (C) The average level of RT signal in enhancer and promoter regions for general dataset. (D-E) PR curves and ROC curves derived from using only the RT feature of enhancer or promoter. (F) Distribution of key features in promoters or enhancers across six cell lines among the top 200 RT features. (G) Frequency of key features distributed in different windows in six cell lines and general model among the top 200 features. A higher frequency indicates a greater contribution of features in that window size to model performance. (H-I) Model performance for both the cell line model and the general model when using RT feature subsets with different windows and RT feature set (Blue line) by integrating all windows. RT: replication timing, AUC/ AUROC: Area Under the Receiver Operating Characteristic; AUPRC: Area Under the Precision-Recall Curve.


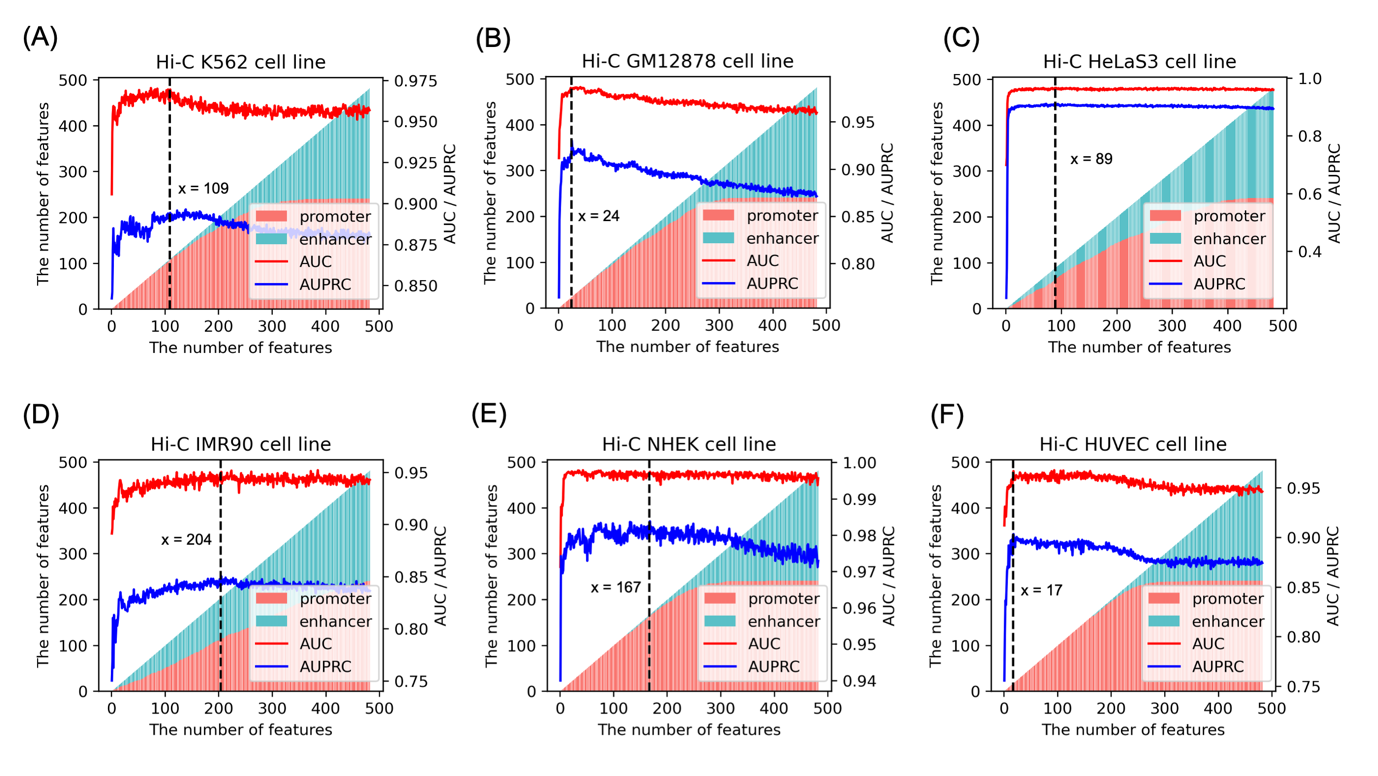


### Figure S8. Importance scores of replication timing (RT) features in promoters and enhancers. In (A) K562, (B) GM12878, (C) HeLaS3, (D) IMR90, (E) NHEK, and (F) HUVEC cell lines. Red and blue lines represent AUC and AUPRC evaluation metrics when performing feature selection based on the incremental feature selection (IFS) rule, respectively.


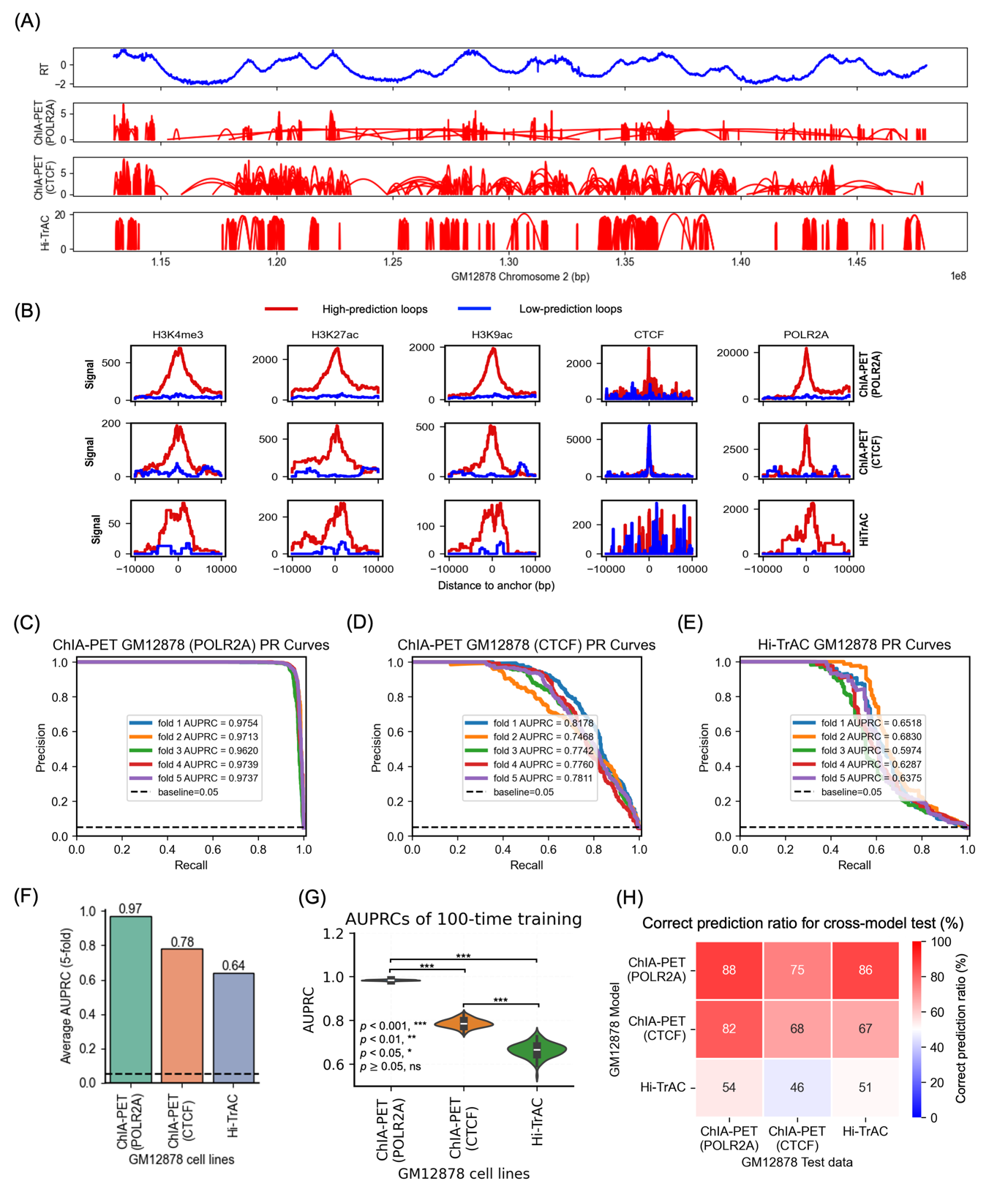


### Figure S9. RepliChrom demonstrates strong cross-platform generalization in predicting chromatin loops. (A) Genomic distribution of RT signals and chromatin interaction loops identified by POLR2A ChIA-PET, CTCF ChIA-PET, and Hi-TrAC on chromosome 2 of the GM12878 cell line. (B) Enrichment of histone modification signals at high and low-predicted loop anchors of POLR2A/ CTCF ChIA-PET and Hi-TrAC, revealing stronger activate epigenetic signals at accurately predicted loops. (C-E) Precision-recall (PR) curves of RepliChrom in 5-fold cross-validation for POLR2A ChIA-PET, CTCF ChIA-PET, and Hi-TrAC data, respectively. AUPRC values demonstrate robust performance across platforms. (F) Average AUPRC values from 5-fold cross-validation for the three platforms. (G) Distribution of AUPRC scores across 100 independent training iterations, highlighting model stability. (H) Cross-platform prediction results: POLR2A/CTCF ChIA-PET and Hi-TrAC models used to predict loops across different platforms, with the percentage of correctly predicted loops shown. AUPRC: Area Under the Precision-Recall Curve; RT: replication timing.

**
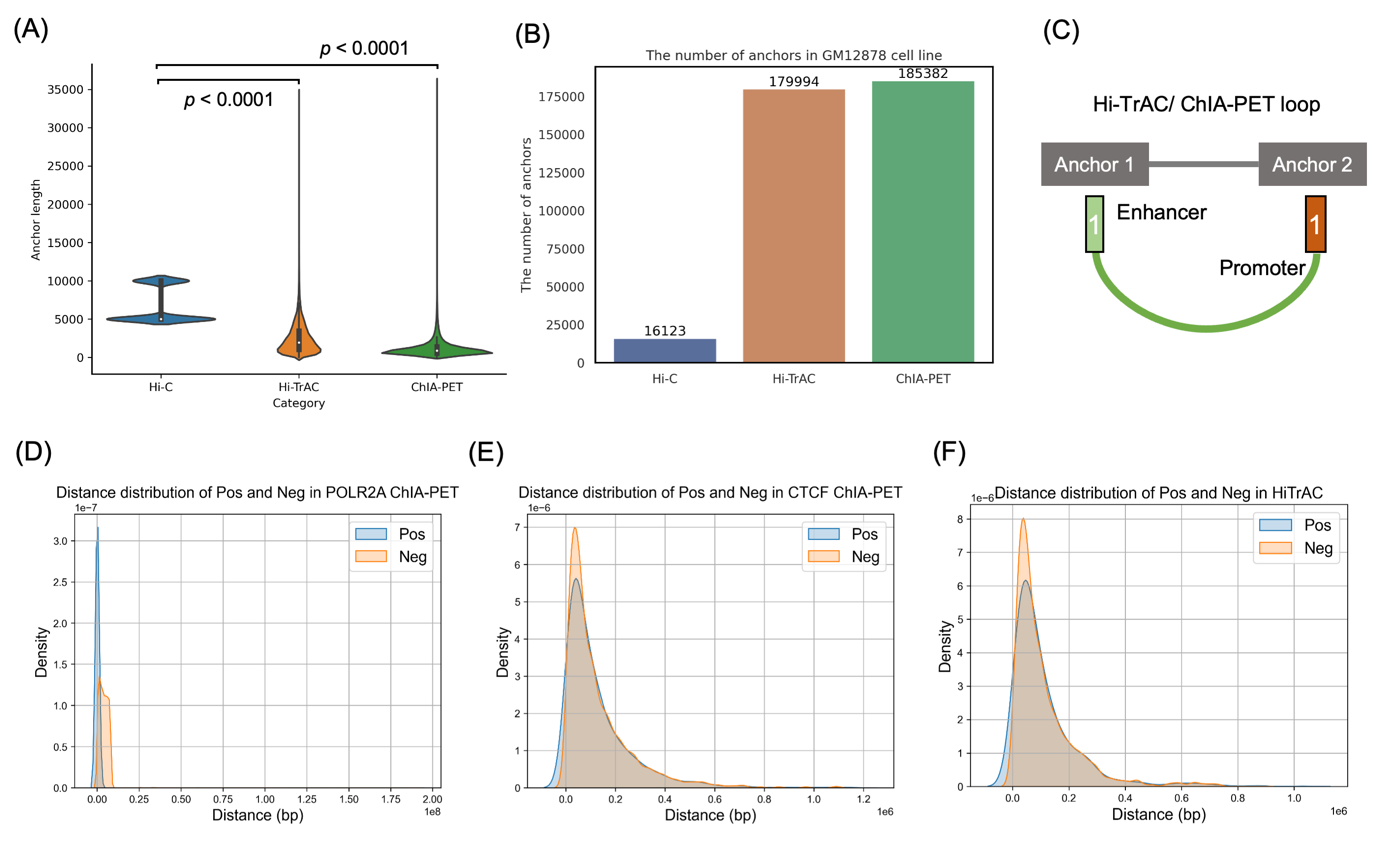
**

### Figure S10. Compare the data of Hi-C, ChIA-PET, and Hi-TrAC. (A) Comparison of anchor length between Hi-C loop anchors and Hi-TrAC/ ChIA-PET loop anchors. (B) The anchor number of Hi-C, Hi-TrAC and ChIA-PET. (C) The positive EPIs extracted from Hi-TrAC and ChIA-PET loop anchor. (D-F) The distance distribution of positive and negative set in POLR2A ChIA-PET, CTCF ChIA-PET, and Hi-TrAC datasets of GM12878 cell line.


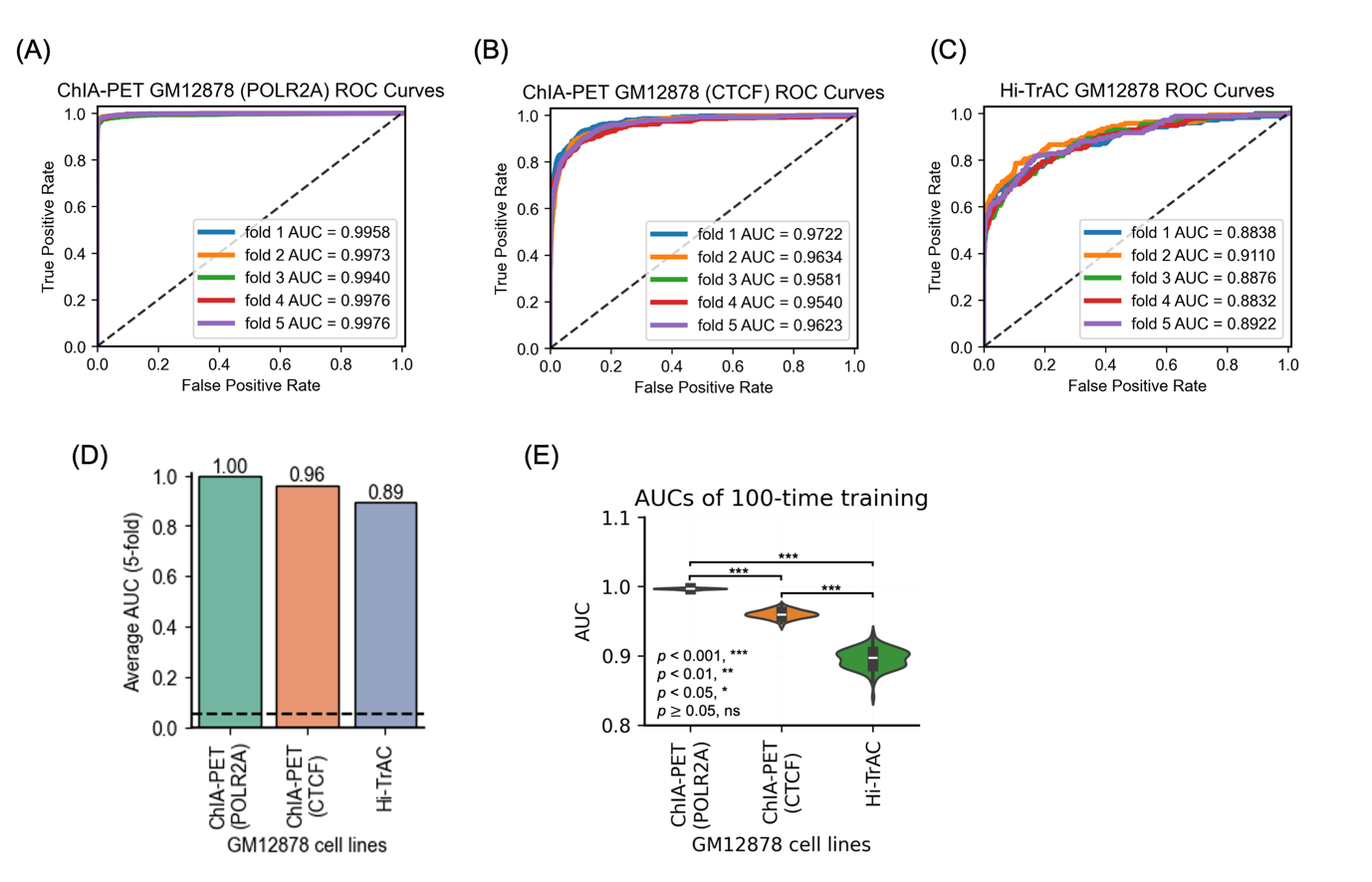


### Figure S11. Evaluation the generalization ability of of RepliChrom in ChIA-PET and Hi-TrAC datasets. (A-C) Performance of the POLR2A ChIA-PET, CTCF ChIA-PET and Hi-TrAC model based on 5-fold cross-validation in GM12878 cell line. (D) The mean AUC values of POLR2A ChIA-PET, CTCF ChIA-PET and Hi-TrAC model based on 5-fold cross-validation in GM12878 cell line. (E) Performance of the POLR2A ChIA-PET, CTCF ChIA-PET and Hi-TrAC model based on 100 times of training and testing in GM12878 cell line.


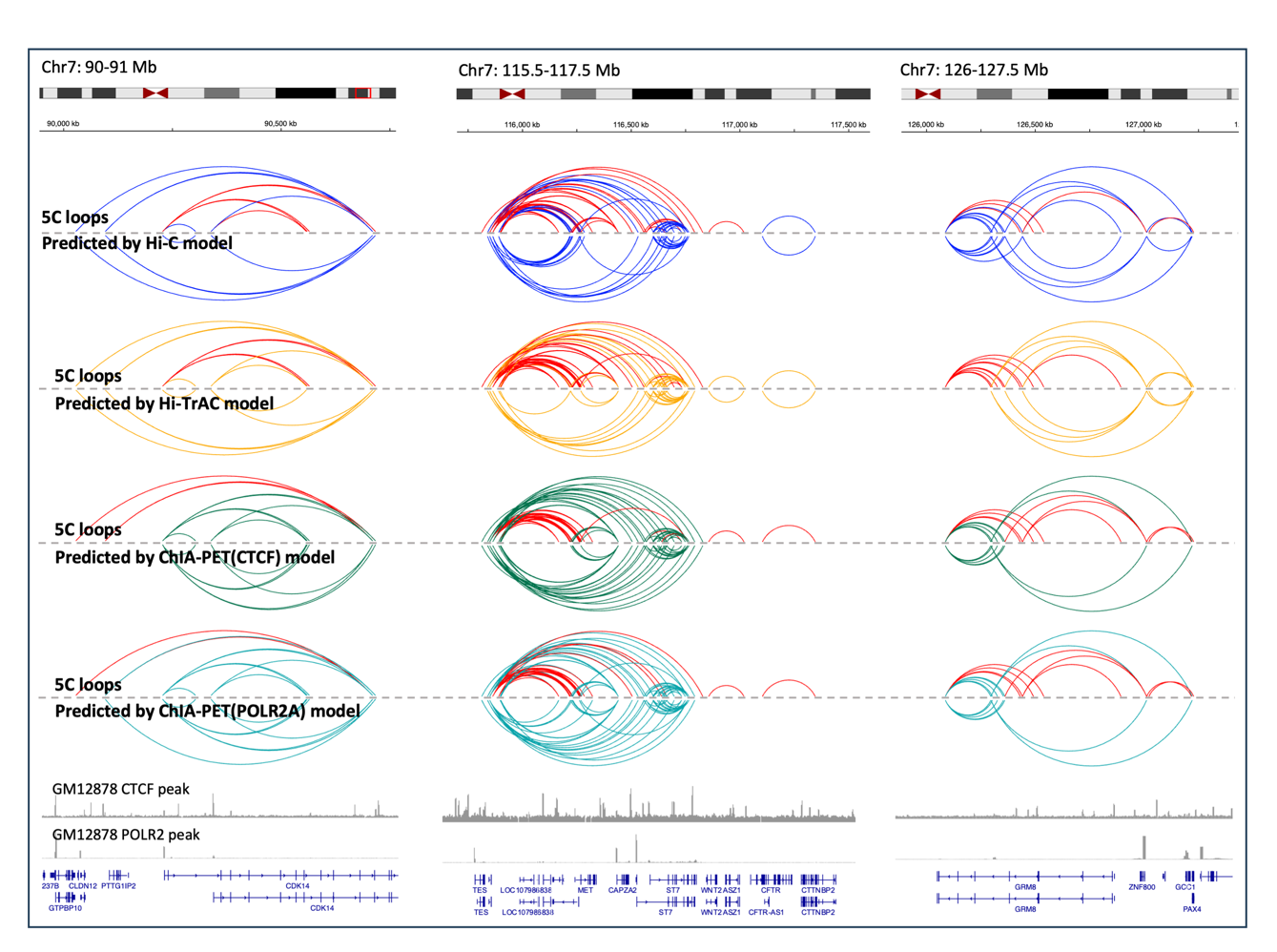


### Figure S12. Validation of RepliChrom predictions using 5C chromatin interaction data. Predicted chromatin interactions compared with true 5C loops in the GM12878 cell line. Colored arcs represent interactions correctly predicted by individual models: Hi-C (blue), Hi-TrAC (orange), ChIA-PET (CTCF, green), and ChIA-PET (POLR2A, cyan). Red arcs indicate 5C interactions missed by each model. 5C: Chromosome Conformation Capture Carbon Copy.


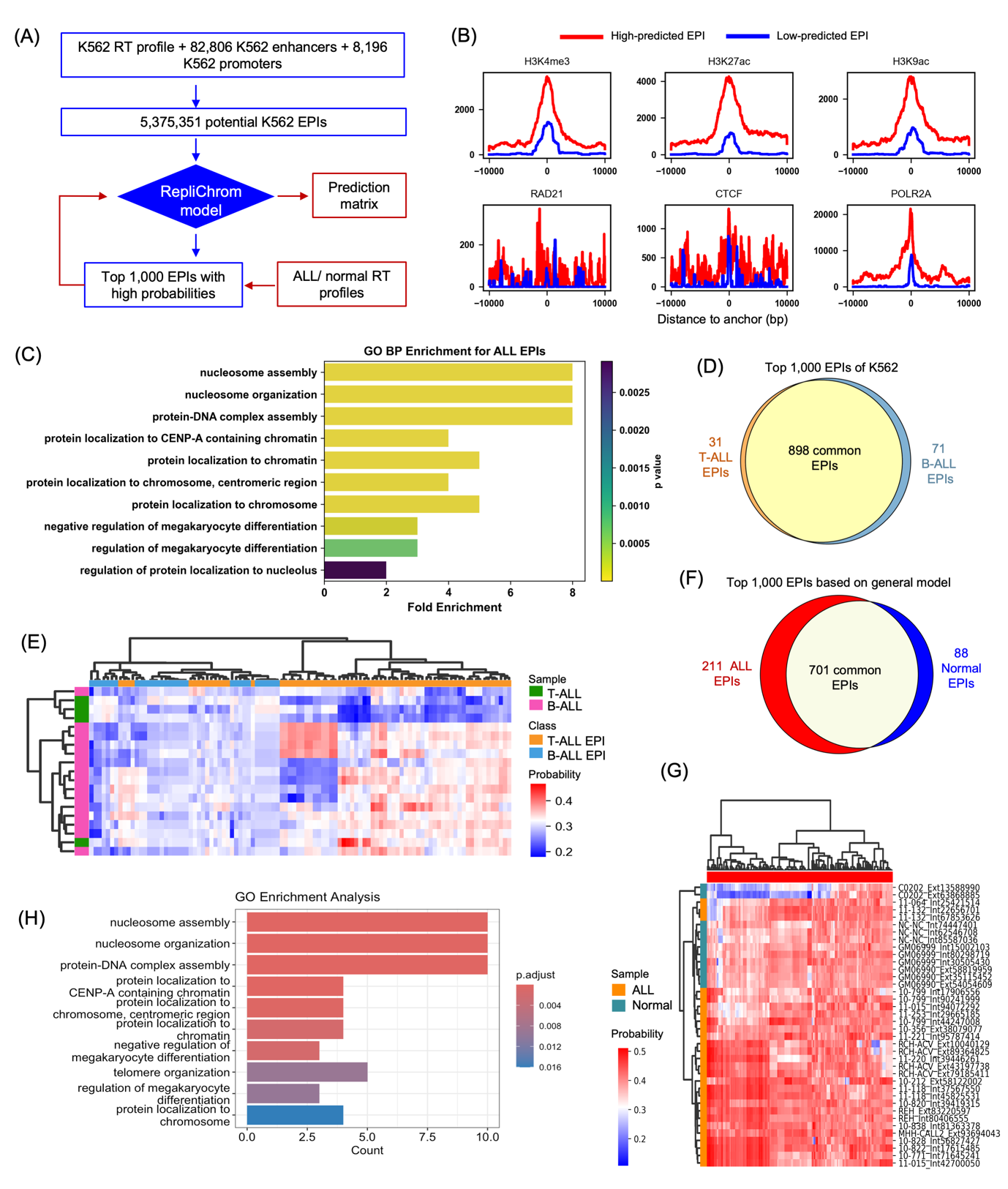


### Figure S13. Model application in acute lymphoblastic leukemia (ALL) samples. (A) Steps of RepliChrom model to mine ALL specific enhancer-promoter interactions (EPIs). (B) Enrichment of histone modifications and transcription factors at high and low-predicted K562 EPIs. (C) Gene ontology (GO) analysis for the 62 genes from the 137 ALL EPIs. (D) Venn diagram showing the T-ALL specific and B-ALL specific EPIs among the top 1000 K562 EPIs. (E) Hierarchical clustering of T-ALL (Green) and B-ALL (Magenta) samples based on the selected T-ALL EPIs (Orange) and B-ALL EPIs (Light blue). The heatmap represents the model prediction probability, with larger values appearing redder. (F) Venn diagram showing the ALL specific and normal specific EPIs among the top 1,000 EPIs predicted by general model. (G) Hierarchical clustering of ALL (Orange) and normal (Cyan) samples based on the selected ALL EPIs (Red). The heatmap represents the model prediction probability. The larger the value, the redder it is. (H) GO analysis for the 76 genes from the 211 ALL EPIs.


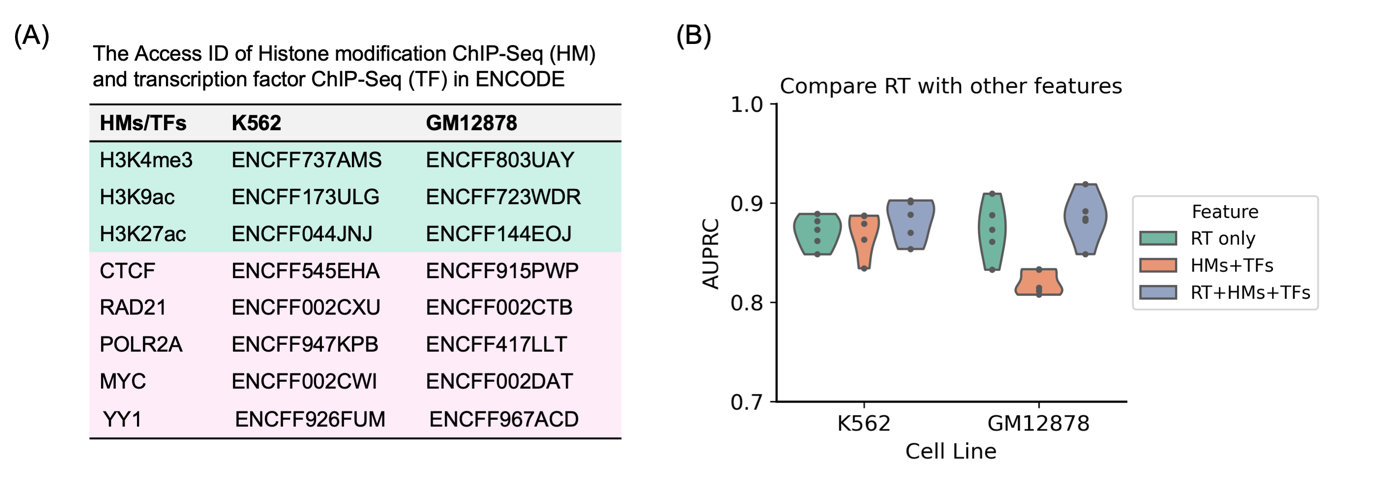


### Figure S14. Compare replication timing (RT) features with epigenetics signals. (A) The data of histone modifications (HMs) and transcription factors (TFs) was collected from ENCODE. (B) The AUPRC values for model trained by different feature sets including RT, HMs+TFs, and RT+HMs+TFs.

## References

1. Weddington, Nodin, Alexander Stuy, Ichiro Hiratani, Tyrone Ryba, Tomoki Yokochi, David M. Gilbert. 2008. “ReplicationDomain: a visualization tool and comparative database for genome-wide replication timing data.” *BMC Bioinformatics* 9: 530. <https://doi.org/10.1186/1471-2105-9-530>

2. Ryba, Tyrone, Dana Battaglia, Bill H Chang, James W Shirley, Quinton Buckley, Benjamin D Pope, Meenakshi Devidas, Brian J Druker, David M Gilbert. 2012. “Abnormal developmental control of replication-timing domains in pediatric acute lymphoblastic leukemia.” *Genome Research* 22: 1833-1844. <https://doi.org/10.1101/gr.138511.112>

3. Ryba, Tyrone, Dana Battaglia, Benjamin D. Pope, Ichiro Hiratani, David M. Gilbert. 2011. “Genome-scale analysis of replication timing: from bench to bioinformatics.” *Nature Protocols* 6: 870-895. <https://doi.org/10.1038/nprot.2011.328>

4. Zhu, Xiaopeng, Yang Zhang, Yuchuan Wang, Dechao Tian, Andrew S. Belmont, Jason R. Swedlow, Jian Ma. 2022. “Nucleome Browser: an integrative and multimodal data navigation platform for 4D Nucleome.” *Nature Methods* 19: 911-913. <https://doi.org/10.1038/s41592-022-01559-3>

5. Wang, Jian, Alton B. Farris, Kaiming Xu, Ping Wang, Xiangming Zhang, Duc M. Duong, Hong Yi, Hui-Kuo Shu, Shi-Yong Sun, Ya Wang. 2016. “GPRC5A suppresses protein synthesis at the endoplasmic reticulum to prevent radiation-induced lung tumorigenesis.” *Nature Communications* 7: 11795. <https://doi.org/10.1038/ncomms11795>

6. Chen, Yulong, Jiong Deng, Junya Fujimoto, Humam Kadara, Taoyan Men, Dafna Lotan, Reuben Lotan. 2010. “Gprc5a deletion enhances the transformed phenotype in normal and malignant lung epithelial cells by eliciting persistent Stat3 signaling induced by autocrine leukemia inhibitory factor.” *Cancer Research* 70: 8917-8926. <https://doi.org/10.1158/0008-5472.CAN-10-0518>

7. Marchal, Claire, Jiao Sima, David M. Gilbert. 2019. “Control of DNA replication timing in the 3D genome.” *Nature Reviews Molecular Cell Biology* 20: 721-737. <https://doi.org/10.1038/s41580-019-0162-y>

8. Solovei, Irina, Moritz Kreysing, Christian Lanctôt, Süleyman Kösem, Leo Peichl, Thomas Cremer, Jochen Guck, Boris Joffe. 2009. “Nuclear architecture of rod photoreceptor cells adapts to vision in mammalian evolution.” *Cell* 137: 356-368. <https://doi.org/10.1016/j.cell.2009.01.052>

9. Rao, Suhas S P., Miriam H Huntley, Neva C Durand, Elena K Stamenova, Ivan D Bochkov, James T Robinson, Adrian L Sanborn, et al. 2014. “A 3D map of the human genome at kilobase resolution reveals principles of chromatin looping.” *Cell* 159: 1665-1680. <https://doi.org/10.1016/j.cell.2014.11.021>

10. Andersson, Robin, Claudia Gebhard, Irene Miguel-Escalada, Ilka Hoof, Jette Bornholdt, Mette Boyd, Yun Chen, et al. 2014. “An atlas of active enhancers across human cell types and tissues.” *Nature* 507: 455-461. <https://doi.org/10.1038/nature12787>

11. Nassar, Luis R., Galt P. Barber, Anna Benet-Pagès, Jonathan Casper, Hiram Clawson, Mark Diekhans, Clay Fischer, et al. 2023. “The UCSC genome browser database: 2023 update.” *Nucleic Acids Research* 51: D1188-D1195. <https://doi.org/10.1093/nar/gkac1072>

12. Ernst, Jason, Pouya Kheradpour, Tarjei S. Mikkelsen, Noam Shoresh, Lucas D. Ward, Charles B. Epstein, Xiaolan Zhang, et al. 2011. “Mapping and analysis of chromatin state dynamics in nine human cell types.” *Nature* 473: 43-49. <https://doi.org/10.1038/nature09906>

13. Dunham, Ian, Anshul Kundaje, Shelley F. Aldred, Patrick J. Collins, Carrie A. Davis, Francis Doyle, Charles B. Epstein, et al. 2012. “An integrated encyclopedia of DNA elements in the human genome.” *Nature* 489: 57-74. <https://doi.org/10.1038/nature11247>

14. Frankish, Adam, Mark Diekhans, Irwin Jungreis, Julien Lagarde, Jane E Loveland, Jonathan M. Mudge, Cristina Sisu, et al. 2021. “GENCODE 2021.” *Nucleic Acids Research* 49: D916-D923. <https://doi.org/10.1093/nar/gkaa1087>

15. Fullwood, Melissa J., Mei Hui Liu, You Fu Pan, Jun Liu, Han Xu, Yusoff Bin Mohamed, Yuriy L. Orlov, et al. 2009. “An oestrogen-receptor-α-bound human chromatin interactome.” *Nature* 462: 58-64. <https://doi.org/10.1038/nature08497>

16. Liu, Shuai, Yaqiang Cao, Kairong Cui, Qingsong Tang, Keji Zhao. 2022. “Hi-TrAC reveals division of labor of transcription factors in organizing chromatin loops.” *Nature Communications* 13: 6679. <https://doi.org/10.1038/s41467-022-34276-8>

17. Tang, Zhonghui, Oscar Junhong Luo, Xingwang Li, Meizhen Zheng, Jacqueline Jufen Zhu, Przemyslaw Szalaj, Pawel Trzaskoma, et al. 2015. “CTCF-mediated human 3D genome architecture reveals chromatin topology for transcription.” *Cell* 163: 1611-1627. <https://doi.org/10.1016/j.cell.2015.11.024>

18. Sanyal, Amartya, Bryan R. Lajoie, Gaurav Jain, Job Dekker. 2012. “The long-range interaction landscape of gene promoters.” *Nature* 489: 109-113. <https://doi.org/10.1038/nature11279>

19. Becker, Thijs, Axel-Jan Rousseau, Melvin Geubbelmans, Tomasz Burzykowski, Dirk Valkenborg. 2023. “Decision trees and random forests.” *American Journal of Orthodontics and Dentofacial Orthopedics* 164: 894-897. <https://doi.org/10.1016/j.ajodo.2023.09.011>

20. Whalen, Sean, Rebecca M. Truty, Katherine S. Pollard. 2016. “Enhancer–promoter interactions are encoded by complex genomic signatures on looping chromatin.” *Nature Genetics* 48: 488-496. <https://doi.org/10.1038/ng.3539>

21. Cao, Qin, Christine Anyansi, Xihao Hu, Liangliang Xu, Lei Xiong, Wenshu Tang, Myth T. S. Mok, et al. 2017. “Reconstruction of enhancer–target networks in 935 samples of human primary cells, tissues and cell lines.” *Nature Genetics* 49: 1428-1436. <https://doi.org/10.1038/ng.3950>

22. Salviato, Elisa, Vera Djordjilović, Judith Mary Hariprakash, Ilario Tagliaferri, Koustav Pal, Francesco Ferrari. 2021. “Leveraging three-dimensional chromatin architecture for effective reconstruction of enhancer–target gene regulatory interactions.” *Nucleic Acids Research* 49: e97-e97. <https://doi.org/10.1093/nar/gkab547>

23. Kai, Yan, Jaclyn Andricovich, Zhouhao Zeng, Jun Zhu, Alexandros Tzatsos, Weiqun Peng. 2018. “Predicting CTCF-mediated chromatin interactions by integrating genomic and epigenomic features.” *Nature Communications* 9: 4221. <https://doi.org/10.1038/s41467-018-06664-6>

24. Cao, Fan, Yu Zhang, Yichao Cai, Sambhavi Animesh, Ying Zhang, Semih Can Akincilar, Yan Ping Loh, et al. 2021. “Chromatin interaction neural network (ChINN): a machine learning-based method for predicting chromatin interactions from DNA sequences.” *Genome Biology* 22: 226. <https://doi.org/10.1186/s13059-021-02453-5>

25. Dao, Fu-Ying, Hao Lv, Fang Wang, Chao-Qin Feng, Hui Ding, Wei Chen, Hao Lin. 2019. “Identify origin of replication in Saccharomyces cerevisiae using two-step feature selection technique.” *Bioinformatics* 35: 2075-2083. <https://doi.org/10.1093/bioinformatics/bty943>

26. Forrest, Alistair R. R., Hideya Kawaji, Michael Rehli, J. Kenneth Baillie, Michiel J. L. de Hoon, Vanja Haberle, Timo Lassmann, et al. 2014. “A promoter-level mammalian expression atlas.” *Nature* 507: 462-470. <https://doi.org/10.1038/nature13182>

27. Ranieri, Roberta, Giulia Pianigiani, Sofia Sciabolacci, Vincenzo Maria Perriello, Andrea Marra, Valeria Cardinali, Sara Pierangeli, et al. 2022. “Current status and future perspectives in targeted therapy of NPM1-mutated AML.” *Leukemia* 36: 2351-2367. <https://doi.org/10.1038/s41375-022-01666-2>

28. McLendon, Roger, Allan Friedman, Darrell Bigner, Erwin G. Van Meir, Daniel J. Brat, Gena M. Mastrogianakis, Jeffrey J. Olson, et al. 2008. “Comprehensive genomic characterization defines human glioblastoma genes and core pathways.” *Nature* 455: 1061-1068. <https://doi.org/10.1038/nature07385>
